# Active Center Control of Termination by RNA Polymerase III and tRNA Gene Transcription Levels *In Vivo*

**DOI:** 10.1371/journal.pgen.1006253

**Published:** 2016-08-12

**Authors:** Keshab Rijal, Richard J. Maraia

**Affiliations:** Intramural Research Program, Eunice Kennedy Shriver National Institute of Child Health and Human Development, National Institutes of Health, Bethesda, Maryland, United States of America; The Ohio State University, UNITED STATES

## Abstract

The ability of RNA polymerase (RNAP) III to efficiently recycle from termination to reinitiation is critical for abundant tRNA production during cellular proliferation, development and cancer. Yet understanding of the unique termination mechanisms used by RNAP III is incomplete, as is its link to high transcription output. We used two tRNA-mediated suppression systems to screen for Rpc1 mutants with gain- and loss- of termination phenotypes in *S*. *pombe*. 122 point mutation mutants were mapped to a recently solved 3.9 Å structure of yeast RNAP III elongation complex (EC); they cluster in the active center bridge helix and trigger loop, as well as the pore and funnel, the latter of which indicate involvement of the RNA cleavage domain of the C11 subunit in termination. Purified RNAP III from a readthrough (RT) mutant exhibits increased elongation rate. The data strongly support a kinetic coupling model in which elongation rate is inversely related to termination efficiency. The mutants exhibit good correlations of terminator RT *in vitro* and *in vivo*, and surprisingly, amounts of transcription *in vivo*. Because assessing *in vivo* transcription can be confounded by various parameters, we used a tRNA reporter with a processing defect and a strong terminator. By ruling out differences in RNA decay rates, the data indicate that mutants with the RT phenotype synthesize more RNA than wild type cells, and than can be accounted for by their increased elongation rate. Finally, increased activity by the mutants appears unrelated to the RNAP III repressor, Maf1. The results show that the mobile elements of the RNAP III active center, including C11, are key determinants of termination, and that some of the mutations activate RNAP III for overall transcription. Similar mutations in spontaneous cancer suggest this as an unforeseen mechanism of RNAP III activation in disease.

## Author Summary

RNA polymerase (RNAP) III is a multisubunit enzyme that synthesizes large amounts of transfer RNAs and a few other abundant short structural RNAs essential for the proliferation of all eukaryotic cells. To meet this demand, RNAP III must repeatedly initiate and terminate RNA synthesis with high efficiency, up to one million times on each tRNA gene per cell doubling. Much more research has been done on initiation than on termination. We performed a genetic screen focused on Rpc1, the largest RNAP III subunit, after subjecting it to random mutagenesis and identifying single amino acid substitutions that specifically affected its ability to terminate. Over 120 mutations were isolated and mapped onto a recently published, near-atomic resolution structure of RNAP III in synthesis mode. Mutations were concentrated in mobile elements of the active center of the enzyme, the bridge helix and the trigger loop, as well as regions that help recruit the RNA 3' cleavage domain of the C11 subunit which transiently interacts with the active center. The data provide evidence that RNAP III controls termination via its active center. Biochemical characterization demonstrated that a mutant RNAP III synthesizes RNA at a higher rate than wild type and also synthesizes more RNA *in vivo*.

## Introduction

The core structures of multisubunit RNA polymerases (RNAP) have been conserved in all domains of life [1]. The 5- and 12- subunit RNAPs of bacteria and archaea synthesize all RNAs while eukaryotes contain three major RNAPs, I, II, and III, with 14, 12 and 17 subunits, respectively, that synthesize different RNAs [2]. Plants also contain RNAPs IV & V, which are specialized homologs of RNAP II [3, 4]. The RNAP active center, which is formed mostly by an interface of the two largest subunits, has a catalytic site at one end of an 8–9 bp RNA:DNA hybrid binding cavity, while the incoming duplex DNA is held in a separate binding cleft [5, see 6]. The catalytic mechanism relies on two mobile elements, the bridge helix (BH) and trigger loop (TL), which position substrate and promote forward movement by a ratchet-like mechanism [7–10]. If RNAP should pause, it remains bound even if the RNA 3' end becomes misaligned with the active center; in this case, the elongation factors GreB (bacteria) or TFIIS (RNAP II) can access the active site through a pore via the funnel entrance, and stimulate endonucleolytic cleavage of the RNA so that its newly created 3' OH end becomes aligned with the catalytic center and synthesis can resume [11, 12]. The RNA:DNA hybrid adds stability to the complex, without which premature dissociation may occur [13, 14]. Indeed, formation of the intrinsically weak base paired hybrid, oligo(rU:dA) [15] which occurs upon transcription of an oligo(dA) template, is a key determinant of termination by RNAP III [16, 17].

Encountering a cis-sequence termination signal initiates RNAP to pause, release the RNA from its active center and dissociate from DNA [18]. The cis-termination signals differ among RNAPs as do the mechanisms involved [19, 20]. The cis signal in DNA for RNAP III is simple and most closely coincides with the site at which RNA synthesis ceases and release occurs [21]. RNAP III terminates at oligo(T), which is found only at the ends of the genes it transcribes, and requires no other cis-elements or ancillary factors [22]. The majority of genes transcribed by RNAP III are for tRNAs, whose production during cell proliferation, development and oncogenesis [23–26] rely on efficient recycling from termination to reinitiation [20]. RNAP III subunits C82/C34/C31, C53/C37 and C11 are homologs of RNAP II initiation factors TFIIE, TFIIF, and elongation factor TFIIS, respectively [27]. C82/C34/C31 is required for initiation, and C53/C37/C11 for termination and facilitated reinitiation [28]. It is noteworthy that the RNA cleavage form of C11 is required for RNAP III reinitiation [28]. Yet, the significance of this activity and of other subunit relationships with termination, reinitiation and transcription output *in vivo* are largely unknown and have only begun to be examined [29].

Biochemical data indicate that RNAP III has two modes of termination, a holoenzyme mode at short tracts of 5–7 Ts and a RNAP III-core mode that requires longer tracts of 8–9 Ts [16, 30]. Efficient termination at 5–7 Ts (as found at most yeast tRNA genes) requires the C37/53/11 termination-reinitiation subcomplex. RNAP III-core, which lacks C37/53/11 can terminate with high efficiency but only at the distal end of a 9T tract [16, 30]. Because C37/53/11 is critical for termination and reinitiation by holo-RNAP III but is absent from core-RNAP III [16, 28, 30, 31], these two modes must use different mechanisms to terminate. Actual tRNA terminators are complex [32], leaving open the possibility that a fraction of RNAP III that reads through the proximal region of a long terminator will terminate in its distal region by a core-like mechanism, with outcome that may differ with regard to recycling, reinitiation and/or another aspect of transcription.

A prior screen in *S*. *cerevisiae* to identify any factor involved in tRNA gene termination uncovered the second largest subunit of RNAP III, *RPC2* [33]. Further analyses suggested a kinetic coupling model that posits that a faster polymerase spends less time at the terminator with less opportunity for termination, and vice versa [34]. Studies of C53/C37 and C11 were consistent with this since their depletion from RNAP III increased elongation rate and readthrough of oligo(T) terminators [28, 30, 31].

RNAP III termination has been examined by genetic and biochemical means in our laboratory [22, 29, 30, 35–37]. Use of suppressor-tRNA reporters in *S*. *pombe* together with random mutagenesis of RNAP III subunits yielded a wealth of mutants [reviewed in 38]. For the C37 and C11 subunits this identified 'hot-spot' regions that associate with the RNAP III active center [29, 36, 37]. Additional data indicate that the oligo(T) terminator harbors separate information in its template and nontemplate strands, and suggest that C37 recognizes the T4 and T5 residues [16]. A cryo-EM structure of a RNAP III EC revealed a C37 C-terminal domain (CTD) with an unstructured loop containing the hot-spot region that is hypothesized to lie in close proximity to the nontemplate DNA during termination [17]. Thus, there is an emerging view that the active center of RNAP III at termination involves features of multiple subunits.

Here we used two genetic screens to uncover termination mutants in the largest RNAP III subunit, Rpc1 (C1). 122 point mutations cluster in the BH and TL, as well as in the pore entrance and funnel, the latter outlining a path to the active center for C11. Increased elongation rate was demonstrated for a multiple-isolate mutant. The results indicate active center mobile elements as the major determinants of RNAP III termination. Surprisingly, our data show that the terminator readthrough mutants synthesize more RNA per tRNA reporter gene *in vivo*. Further analysis indicate that this is unrelated to the RNAP III repressor, Maf1. Thus, this work revealed activating mutations that can be rate-limiting for transcription *in vivo*. Notably, a frequently isolated TL activating mutant bears a mutation at the same conserved position as recently reported for one of a set of human RPC1 mutants in spontaneous human cancer [39].

## Results

### The genetic screen

Termination efficiency of *S*. *pombe* RNAP III at the minimal terminator, 5 Ts is ~90% but is negligible at 4 Ts [29, 35, 37]. Our laboratory developed different suppressor-tRNA (sup-tRNA) alleles as reporters for different parts of the tRNA biogenesis pathway, including transcription termination [29, 35–37] [reviewed in 38]. For this study, *S*. *pombe* Rpc1 was subjected to genetic screens for two types of mutants, decreased termination (loss-of-function, LOF) at an otherwise efficient 5T terminator of a tRNA gene, and increased termination (gain-of-function, GOF) at an otherwise suboptimal 4T terminator. The sup-tRNA genes used for the two screens were designed differently so that the LOF and GOF mutants each produce a positive activity that leads to a colony with a white suppression phenotype amongst a background of red colonies. We used *S*. *pombe* strain yKR1 which carries a dimeric tRNA allele to screen for LOF mutants that read through a tRNA gene 5T terminator. yKR1 colonies are normally red because the *ade6-704* allele contains a premature stop codon. If produced, the sup-tRNA will decode the stop codon to make functional Ade6 enzyme and the colony appears white. In yKR1 the sup-tRNA lies downstream of the 5T terminator of an upstream tRNA sequence [29]. RNAP III must initiate at the upstream tRNA; the suppression phenotype occurs if a mutant RNAP III reads through its 5T terminator to transcribe the downstream sup-tRNA which is followed by a 21T failsafe terminator [29].

As was done previously [29, 36, 37], the screens were performed in the presence of the chromosomal wild type allele, in this case *rpc1*^***+***^, by transforming the test strains with plasmid libraries of mutagenized C1 alleles. This approach can theoretically uncover mutations that might otherwise be lethal in the absence of the wild type protein.

**Fig 1:**
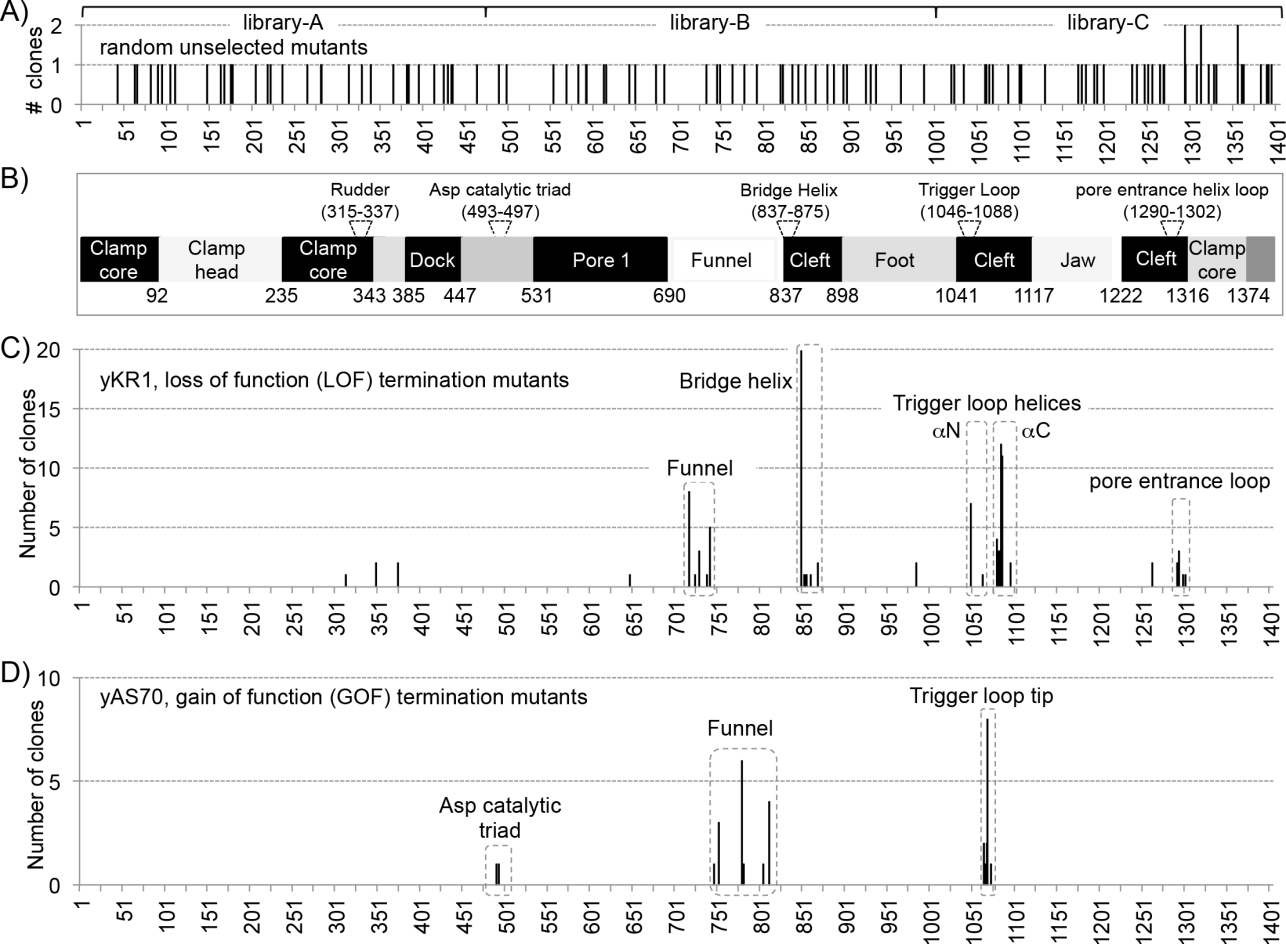
Distribution of point mutations in *S*. *pombe* RNAP III C1 termination mutants. **A)** Unselected randomly chosen bacterial colonies. **B)** Linear schematic representation of *S*. *pombe* Rpc1 (C1), based on sequence homology to *S*. *cerevisiae* C160 [17]. **C)** Mutations in LOF termination (readthrough) mutants selected in yKR1. **D)** Mutations in GOF termination mutants selected in yAS70.

The *rpc1*^***+***^ gene is ~4.5 kb, and the encoded protein is 1405 amino acids. Three libraries were made, each with mutations in only one-third of the *rpc1*^***+***^ length; 1–480, 480–1006 and 1006–1405, referred to as lib-A, -B, and -C, respectively. Sequencing of ~130 random plasmids prior to screening revealed that the libraries had similar mutation rates (~1/330 amino acids, Table 1) throughout (Fig 1A).

**Table 1:** Library screening of spRpc1.

Library	Mut. Rate	Screening strain	screen number x10^3^	Suppress Rate	Single mutation	Two mutns	Multiple mutns	Suppress strength
Lib A	1/300	yKR1 5T,LOF^1^	500	0.01%	5	6	17	weak
		yJI1 6T,LOF	ND	ND	ND	ND	ND	ND
		yAS70 4T,GOF^2^	500	none	none	none	none	none
Lib B	1/385	yKR1 5T,LOF	180	0.3%	47	33	18	strong
		yJI1 6T,LOF	150	0.005%	3	5	1	strong
		yAS70, 4T,GOF	450	0.009%	19	12	13	medium
Lib C	1/285	yKR1 5T,LOF	150	1.0%	52	29	19	strong
		yJI1 6T,LOF	200	none	none	none	none	none
		yAS70, 4T,GOF	390	0.01%	15	12	11	medium

^**1**^LOF = loss of function

^**2**^GOF = gain of function

### C1 termination LOF mutations map to the BH, TL, pore entrance and funnel

Single mutations are most informative; yKR1 (LOF) yielded 97 mutants and 96% of these were at residues that are identical in *S*. *cerevisiae* RPC1 (Table 2). The five from lib-A showed weak suppression. Lib-B produced 47, clustered in two regions, the funnel and BH (Table 2, Fig 1A–1C), and multiple isolates of the same position were obtained for both. Mutations of E850 were isolated 20 times, more than at any other position (Table 2).

**Table 2:** Single mutation LOF C1 mutants (from yKR1), phenotypes in yKR1, yJI1.

Mutation *S*. *pombe*	Number isolates	Phenotype yKR1 (5T)	Phenotype yJI1 (6T)	*S*. *cerevisiae* C160 pos.	Region (motif/domain)
Y314D	1	Moderate	None	Y326	Clamp core
N349D	2	Moderate	None	N368	Rudder adjacent
A375T	2	Moderate	None	A393	Rudder adjacent
V648A	1	Strong	None	V668	Pore 1
S718P	8	Strong	None	C738	Funnel
Y725D	1	Weak	None	F745	Funnel
L730P	3	Strong	None	L750	Funnel
E739K	1	Weak	None	E759	Funnel
L742P	5	Moderate	None	L762	Funnel
E850G	16	Strong	Strong	E870	BH
E850K	4	Strong	Strong	E870	BH
V853L	1	Moderate	None	V873	BH
D854N	1	Weak	None	D874	BH
A856V	1	Moderate	None	A876	BH
E861G	1	Weak	None	E881	BH
L869V	1	Strong	None	L889	BH
L869F	1	Moderate	None	L889	BH
L985P	2	Strong	None	L1029	Foot
A1049V	4	Strong	Weak	A1091	TL αN
A1049T	1	Strong	None	A1091	TL αN
A1049D	1	Strong	None	A1091	TL αN
T1063A	1	Moderate	None	T1105	TL
V1080A	4	Moderate	None	V1123	TL αC
R1082H	3	Moderate	None	R1125	TL αC
I1083S	1	Moderate	None	I1126	TL αC
I1083T	1	Moderate	None	I1126	TL αC
E1085G	6	Strong	Weak	E1128	TL αC
E1085A	1	Strong	Weak	E1128	TL αC
E1085D	3	Moderate	None	E1128	TL αC
I1086T	9	Strong	None	I1129	TL αC
I1086M	1	Strong	None	I1129	TL αC
P1096L	1	Moderate	None	P1139	Cleft
N1263D	1	Moderate	None	N1317	Cleft pore
N1263S	1	Weak	None	N1317	Cleft pore
H1292R	2	Strong	None	H1346	pore entrance loop
L1294P	2	Moderate	None	M1348	pore entrance loop
R1299Q	1	Strong	None	R1353	pore helix
M1302V	1	Moderate	None	Q1356	pore helix
**Total:**	**97**				

Lib-C yielded 41 single mutants. Mutations in the TL were in the N- and C-terminal α-helixes on either side of the less structured loop tip [7, 8], notably with none in the tip itself. Six were at position 1049 and ten each at 1085 and 1086, substituted with three amino acids each (Table 2). Others clustered at 1263–1302, homologous to the cleft-pore entrance loop (CPEL) between α46 and α47 of Rpb1 (RNAP II) that interacts with residues in the β3 strand of the acidic hairpin CTD of TFIIS [40] which are conserved in the highly homologous CTD of C11 [36]. The BH, TL, funnel and CPEL were the most LOF termination-sensitive regions of C1 (Fig 1C).

### Gain-of-function increased termination mutants do not overlap with LOF mutations

We developed a screen for GOF termination in yAS70 using a monomeric sup-tRNA followed by 4T tract which is normally inadequate for termination. The mutants clustered adjacent to but nonoverlapping with the LOF mutants in the TL and funnel (Fig 1D, Table 3). The GOF mutants in the TL including H1068Q were strictly limited to the loop tip (Fig 1D). In yeast RNAP II, the H that is homologous to *S*. *pombe* C1 H1068 makes contact with the β-phosphate of the incoming NTP and is critical for catalysis [8]. The GOF funnel mutants were nonoverlapping with the LOF funnel mutants (Fig 1C and 1D).

**Table 3:** Single mutation GOF C1 mutants (from yAS70).

Mutation *S*. *pombe*	Number of isolates	*S*. *cerevisiae* C160 position	Region (motif)
N491D	1	N409	catalytic Asp triad
F494L	1	F412	catalytic Asp triad
S747P	1	G767	Funnel
V753A	3	V773	Funnel
K780R	1	K800	Funnel
S782P	1	S802	Funnel
D805G	1	D825	Funnel
L812P	1	L832	Funnel
K1065N	2	K1107	TL tip
T1066I	1	T1108	TL tip
F1067S	1	F1109	TL tip
H1068Q	2	H1110	TL tip
F1069L	7	F1111	TL tip
F1069S	1	F1111	TL tip
A1073T	1	A1115	TL tip
**Total**	**25**		

Two other GOF mutants, N491D and F494L were in the invariant YNADFDGD catalytic aspartate triad motif shared by all multisubunit RNAPs [41]. The homologous N in yeast RNAP II was observed to contact the 3'-OH of the incoming NTP [8].

Based on high conservation of the GOF mutations in the TL and aspartate triad motif and the known functions of the homologous residues, the data suggest that these mutations would impair catalysis and slow polymerization, fitting a kinetic coupling model of increased termination. The GOF mutants grew slowly relative to wild type. Therefore, most but not all of the work described hereafter focused on the LOF mutants.

### Structure-function homology mapping of the LOF and GOF mutations support the kinetic coupling model of termination by RNAP III

**Fig 2:**
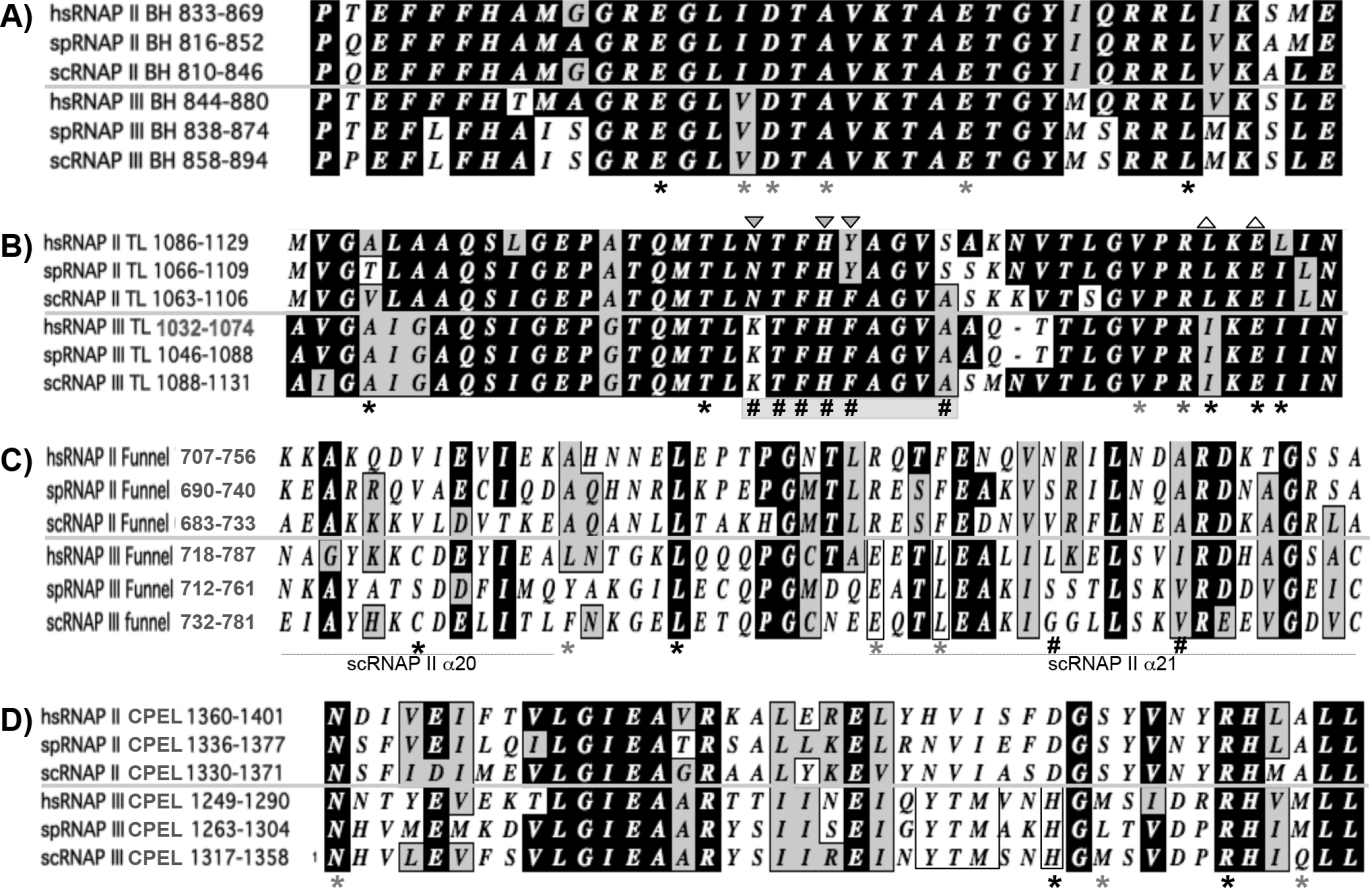
C1 mutants cluster map to highly conserved RNAP motifs. **A-D)** Sequence alignments of homologous regions of RNAP II and III largest subunits of three species, *Homo sapiens* (hs), *Schizosaccharomyces pombe* (sp) *and Saccharomyces cerevisiae* (sc); the bridge helix (BH, A), trigger loop (TL, B), funnel (C) and cleft-pore entrance loop (CPEL, D); black and grey asterisks indicate positions of strong and weaker phenotypes of LOF mutants respectively; # indicates GOF mutations. Upward triangles were placed above residues which when mutated in *S*. *cerevisiae* RNAP II caused increased elongation rate, and downward triangles were placed above residues which when mutated in *S*. *cerevisiae* RNAP II caused decreased elongation rate as summarized in figure 3 of Kaplan et al [42].

**Fig 3:**
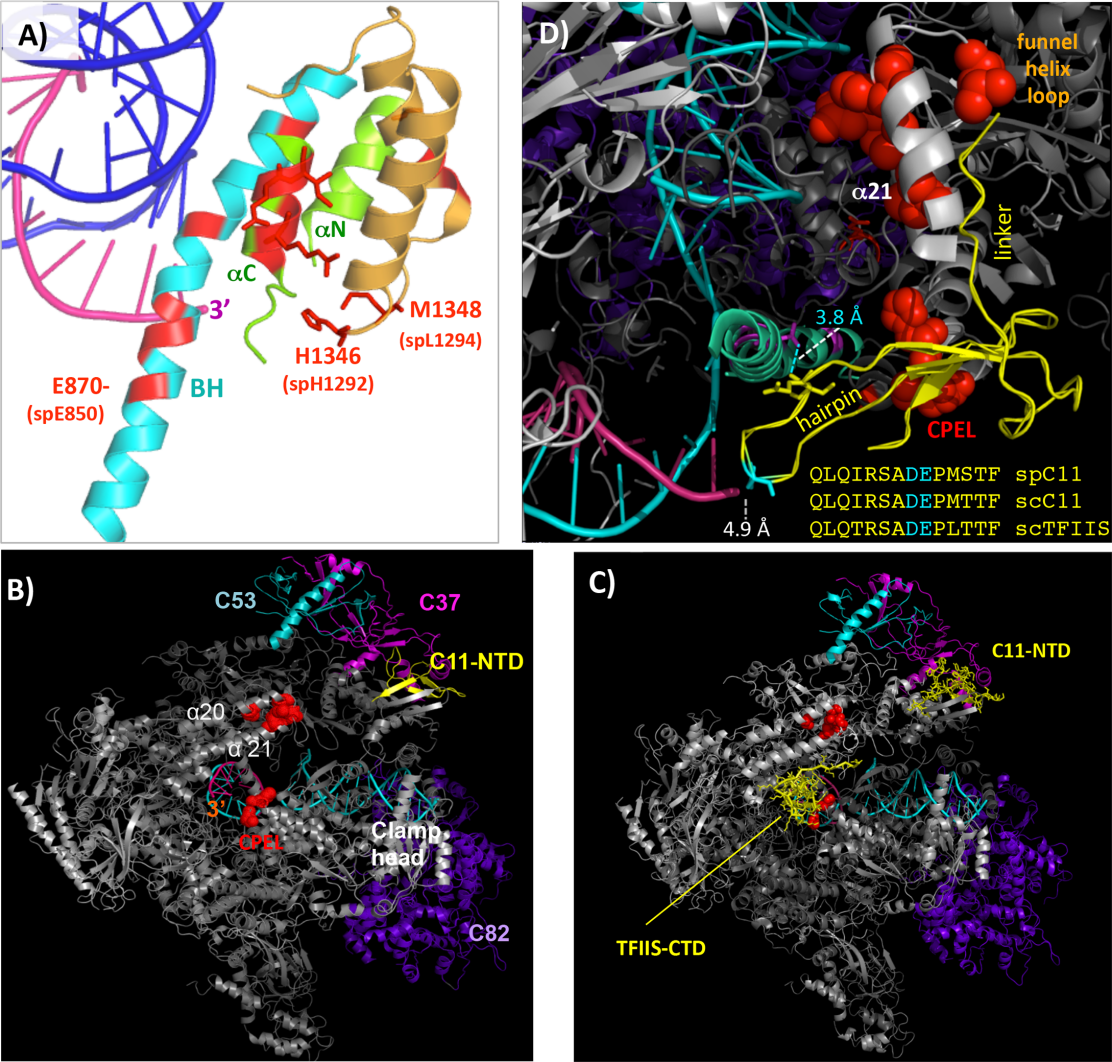
Mapping *S*. *pombe* C1 active center LOF mutants onto *S*. *cerevisiae* RNAP III structure. **A)** Active center bridge helix (BH, cyan) and trigger loop (TL, green) as well as the cleft-pore entrance loop (CPEL, brown) [17]; numbers indicate *S*. *cerevisiae* positions (with *S*. *pombe* positions in parentheses). **B)** The holoenzyme EC structure with mutations in the funnel and CPEL highlighted as red spheres. α20 and α21 refer to RNAP II motifs; CPEL: cleft-pore entrance loop (see text); other subunits and structural features are indicated. The PDB ID used is 5FJ8 [17]. **C)** RNAP III EC structure (PDB 5FJ8) into which the *S*. *cerevisiae* TFIIS CTD and linker was placed (shown in sticks and ribbon backbone mode). **D)** A high resolution view of the acidic hairpin CTD of TFIIS with its linker placed into the RNAP III cryo-EM structure shown in C. The inset shows a sequence alignment of the acidic hairpin regions of the CTDs of C11 and TFIIS from *S*. *cerevisiae* (sc) and *S*. *pombe* (sp), as indicated. The acidic DE residues at the tip of the hairpin are colored cyan to match their stick representation in the structure placement model, which come within 4.9 Å of the RNA 3' end (magenta). Another close contact of 3.8 Å between a C1 residue found mutated in mutant D854N and an invariant Q in TFIIS (the second Q in the sequence alignment) is also indicated. The CPEL, funnel helix loop and α21 are also indicated.

The major mutated regions in the BH and TL region mutants were aligned with homologous regions of RNAPs II and III (Fig 2A & 2B), as were the funnel and CPEL regions (Fig 2C & 2D). Multiple of our mutants were at positions in the TL that when mutated in *S*. *cerevisiae* RNAP II altered elongation rate (triangles above sequences in Fig 2B) [42]. Fifteen of our twenty-five GOF mutants were in the TL and twelve of these were at three positions whose mutated homologs in Rpb1 decreased elongation rate (Table 3, downward triangles in Fig 2B) [42]. Conversely, seventeen of our thirty-six TL LOF mutants were at two positions whose mutated homologs in RNAP II largest subunit increased elongation rate (Table 2, upward triangles in Fig 2B) [42]. That our GOF and LOF mutants exhibit high concordance with previously reported homologous mutants that displayed decreased and increased elongation rate respectively, provide additional strong support to the kinetic coupling model of termination by RNAP III. The active center BH and TL LOF mutations were mapped onto the cryo-EM structure of *S*. *cerevisiae* RNAP III in Fig 3A.

### Evidence of involvement of the RNA cleavage domain of C11 in termination

We aligned the mutated positions in the funnel and CPEL (Tables 2 & 3) with homologous regions of RNAPs II and III (Fig 2C & 2D). The mutations overlap with and in some cases are at homologous residues in RNAP II involved in contacts with the RNA cleavage domain of TFIIS (Fig 2C & 2D, Discussion). C1 mutations to surfaces in the funnel and pore entrance whose RNAP II homologs in Rpb1 interact with the TFIIS CTD which bears its RNA 3' cleavage domain that extends into the active center [40]. Specifically, the funnel helices and their connecting loop that were mutated in our mutants are homologous to Rpb1 α20 and α21 helices (Figs 2C and 3B), the latter of which have extensive contact with TFIIS domain II (linker) and the beginning part of the TFIIS CTD [40]. Mutations to H1292 and L1294, the *S*. *cerevisiae* homologs of which are H1346 and M1348 (Fig 3A and 3B), are on a short loop that face the funnel helices and pore entrance, termed the cleft pore entrance loop (CPEL). This loop appears to be at the same position where the CTD of TFIIS and the homologous A12.2 subunit of RNAP I turn and enter the cleft of their respective RNAP [40, 43]. In RNAP II this CPEL interacts with side chains on the β3 strand of the C-terminal acidic hairpin of TFIIS [40] which are conserved in C11 [36]. The clustering of these functional mutations suggest C11 CTD involvement in termination.

The C11 linker and its CTD were not resolved in the cryo-EM RNAP III elongation structure, presumably due to mobility. However, as can be appreciated in the view in Fig 3B, the first part of the linker coming from the C11 NTD appears as if it may be directed toward the funnel and CPEL, analogous to the linker and homologous CTD of TFIIS [17]. A void is observed from the C11 NTD to the funnel and CPEL as potential space likely occupied by the unresolved linker and CTD of C11 (Fig 3B, C1 funnel and CPEL mutations as red spheres). The funnel helices homologous to Rpb1 α20 and α21, whose connecting loop points toward the C11 NTD, was a hot-spot for mutations (Table 2, Fig 3B, red spheres). Because the CTD sequences of TFIIS and C11 are highly homologous [36], these data provide independent evidence to suggest, and support prior evidence of involvement of the C11 CTD during termination (Discussion).

In further support of such involvement, the *S*. *cerevisiae* RNAP III structure supported excellent placement of the TFIIS CTD structure (Fig 3C). This was not surprising because the TFIIS and C11 CTDs share nearly 90% sequence identity in their acidic hairpin regions [36], and the RNAP II and III core structures are highly homologous including in amino acid sequence of the two largest subunits, and can be superimposed with great fit [2, 44]. An important example of the fit of the TFIIS CTD placement into the RNAP III EC structure is that one of our mutants, D854N represents an invariant position in the largest subunits of RNAPs I, II and III, which in *S*. *cerevisiae* RNAP II is in close contact with the TFIIS invariant Q285 which is also invariant in C11 [36, 40]. In the TFIIS-RNAP III placement model in Fig 3D, the distance between the side chains of these invariant residues is 3.8 Å. This not only provides evidence of a good fit for the placement model but also suggests that the C1 D854N LOF mutation compromises this interaction with the RNA cleavage hairpin of C11 and that this may underlie its termination phenotype.

### The LOF mutants exhibit varying phenotypic strengths

**Fig 4:**
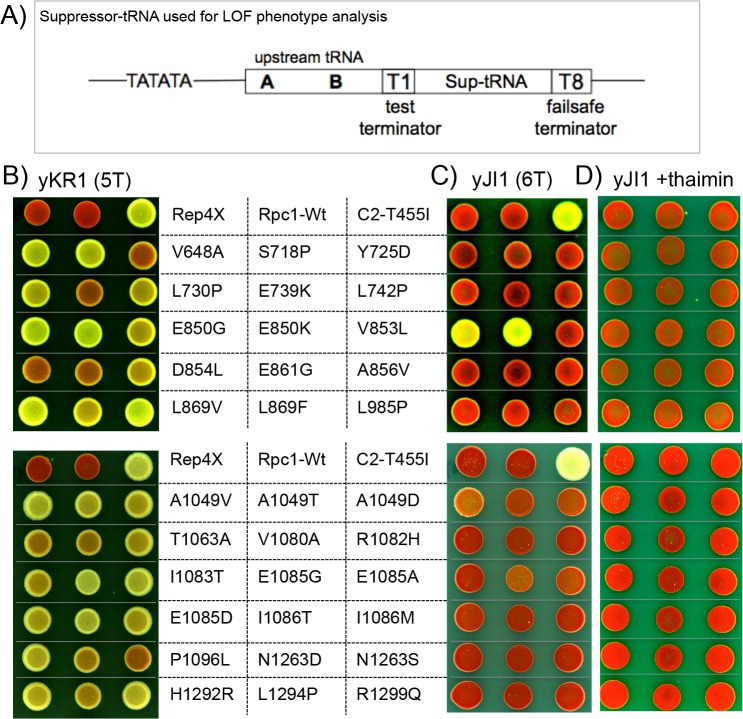
Suppression phenotype strengths of C1 LOF readthrough mutants. **A)** schematic of tRNA reporter allele used for LOF screen and to subsequently examine individual C1 mutants in panels B and C which differ in the number of Ts at the test terminator. **B-D)** Phenotypes of LOF mutants after transformation of recovered plasmids into yKR1 (T5 terminator, B), yJI1 (6T terminator, C) and yJI1 in the presence of thiamin which represses the plasmid promoter, D). Brightest = strongest, darker = weaker. Top line: Rep4X and Rpc1-Wt are negative controls; C2-T455I is a positive control (see text).

Transformation of C1 alleles into yKR1 (Fig 4A and 4B, 5T test terminator) produced a range of suppression strength (degree of whiteness) relative to the negative and positive controls, empty vector Rep4X, C1 wild-type (WT), and a prior characterized mutant in C2, Rpc2-T455I (Fig 4B top row). The mutant alleles were then examined in yJI1 (Fig 4C) whose upstream tRNA bears a stronger, 6T, test terminator. The E850 mutants produced suppression in yJI1 and others produced less (Fig 4C). By this analysis the BH mutants, E850 were strongest, followed by TL mutants A1049 and E1085. Suppression was repressed by thiamin indicating that expression of Rpc1 is required for the phenotype and validating the assay (Fig 4D).

We screened lib-B in yJI1; all mutants had mutation at E850 (Table 1). We made site-directed E850 mutations; all except F, Y and P suppressed yKR1 (S1 Fig).

### The LOF mutant RNAPs III exhibit terminator readthrough *in vitro*

**Fig 5:**
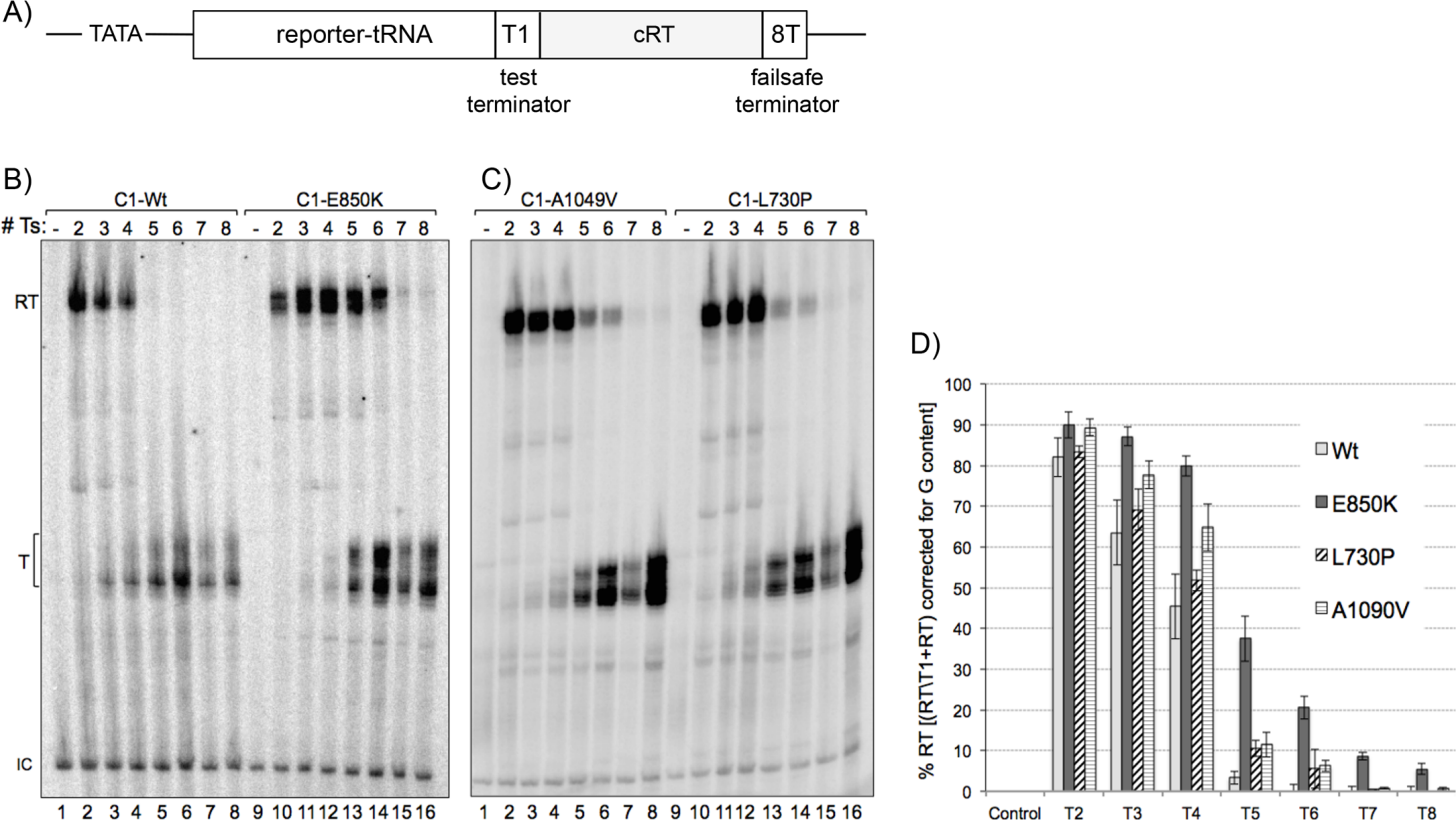
Promoter-dependent *in vitro* transcription reveals oligo(T) length-dependent terminator readthrough by RNAP III C1 mutants. **A)** Schematic of tRNA gene arrangement used for *in vitro* transcription by S100 extracts; the plasmids used for transcription differed only in the number of Ts at the test terminator, T1, as listed above the lanes of B & C. **B-C)** S100 extracts from yKR1-C1 mutants or -C1-Wt control were used as a source of initiation factors and RNAP III as indicated above the lanes. Plasmids containing tRNA genes that differ only in the number of Ts in the oligo(T) terminator as indicated above the lanes were used as templates to program the transcription reactions. RT = readthrough; termination at the failsafe terminator, T = termination at the test terminator, IC = internal control. **D)** Quantitation of % readthrough as defined on the Y-axis; reactions contained 32P-αGTP.

Extracts from *S*. *pombe* yKR1 mutants were used for promoter-dependent transcription of *S*. *pombe* tRNA genes that differ only in the length of their oligo(T) terminators (#Ts; 2T-8T, Fig 5A). While some of the reactions varied in transcription output reflecting plasmid concentration, termination efficiency was internally controlled by the ratio of RT to T. RNAP III C1-Wt transcribed beyond the 2T-4T test terminators as expected but terminated efficiently at 5T-8T (Fig 5B lanes 2–8) also as expected [35]; transcript heterogeneity at T is consistent with pre-tRNA processing in extracts [45]. C1-E850K read past 5T and 6T tracts (Fig 5B lanes 10–16) consistent with their phenotypes. Mutants L730P and A1049V showed more RT than C1-Wt (Fig 5C). Quantitation of % readthrough = RT/T1+RT showed that C1-E850K was the strongest RNAP III mutant, with nearly 40% RT of the 5T terminator (Fig 5D). These data generally fit with the relative phenotypic strengths, with E850 being strongest. We note that C1-Wt RT of T5 was ~5% but decreased to ≤1% at T6-T8, consistent with *in vivo* reporter data [29, 35, 37]. More noteworthy is that even for the strongest mutant, RNAP III-E850K, only ~5% could read through 8T (Fig 5D). We have been unable to isolate any RNAP III mutant including by directed and compound approaches that can read through 8T with significant efficiency (Discussion).

### Elongation by RNAP III C1-mutant is faster than WT

**Fig 6:**
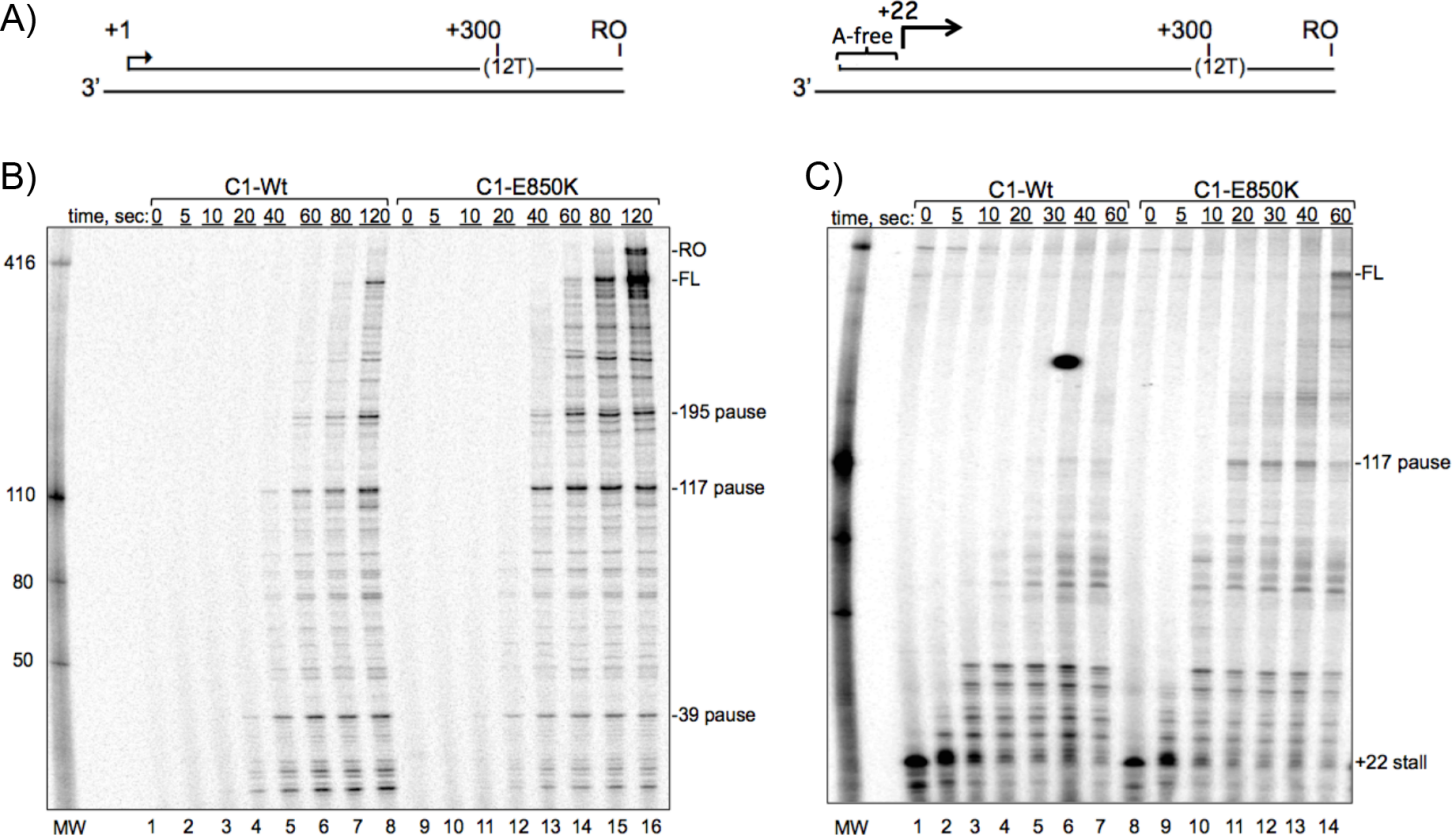
Representative RNAP III C1-E850K mutant exhibits increased elongation. **A)** Schematic of 3'-tailed templates used for promoter-independent transcription by FLAG-purified RNAPs III (see S2 Fig for establishment of assay). The template design on the left was used for panel B and the design on the right was used for panel C. FL = full length 300 nt transcript resulting from termination at 12T, RO = run-off transcript. **B)** Time course of elongation by purified RNAPs III C1-Wt and C1-E850K as indicated above the lanes. **C)** RNAP III was stalled at position +22 followed by a synchronized chase time course of transcription elongation (see text). (The blemish between lanes 5 & 6 is not a transcription signal.)

Strain yKR22 which carries a *rpc2-flag* allele in place of chromosomal *rpc2*^***+***^ was transformed with C1-Wt or C1-E850K and the RNAPs III were purified using anti-Flag-Agarose beads. The RNAPs were used for transcription of a 3'-tailed template with a 12T terminator to produce a full length (FL) transcript of ~300 nt (Fig 6A) in an established RNAP III transcription termination assay (S2 Fig).

Fig 6B shows 0–120 second reactions containing [α-32P]GTP for C1-Wt and C1-E850K RNAPs III. Because transcription was not limited to a single round one can assess relative elongation rates by the time it takes for the RNAPs to first reach the FL site or another site along the way. C1-Wt first reached FL at 80 sec. but this was more clear at 120 sec, while C1-E850K first reached FL at 60 sec. and more clearly at 80. This relative pattern was also seen for a pause site at ~195 nt, as well as earlier pauses (Fig 6B). This suggested that C1-E850K RNAP exhibits faster elongation than C1-Wt RNAP. C1-E850K reproducibly produced more FL transcripts at later times than did C1-Wt despite attempts to equalize the amounts of RNAP III. These data are consistent with C1-E850K having increased elongation rate relative to C1-Wt, terminating at the oligo(T) site, and recycling for additional rounds of transcription.

We designed another tailed template to stall the RNAPs due to lack of a NTP and then compare their elongation rates after synchronized release. A 3'-tailed template that lacks dA in the first 22 nucleotides allowed RNAPs to be elongated in the presence of ATP, CTP and GTP, and stalled at +22, then chased upon addition of UTP. As seen in Fig 6C, the relative amounts of C1-Wt and C1-E850K at +22 (lanes 1 & 8) were comparable. After addition of UTP the RNAPs elongated their transcripts at different rates. C1-E850K reached the 117 pause site and the FL site faster than C1-Wt. Thus, C1-E850K exhibits faster elongation than C1-Wt, as expected of the kinetic coupling model of transcription termination. C1 LOF mutants at E850 were isolated more frequently than any others. The data suggest that these BH and the TL mutations promote faster catalysis and/or translocation, perhaps because these mutated motifs themselves allow more dynamic movement.

### The C1 mutants bypass tRNA terminators *in vivo*

**Fig 7:**
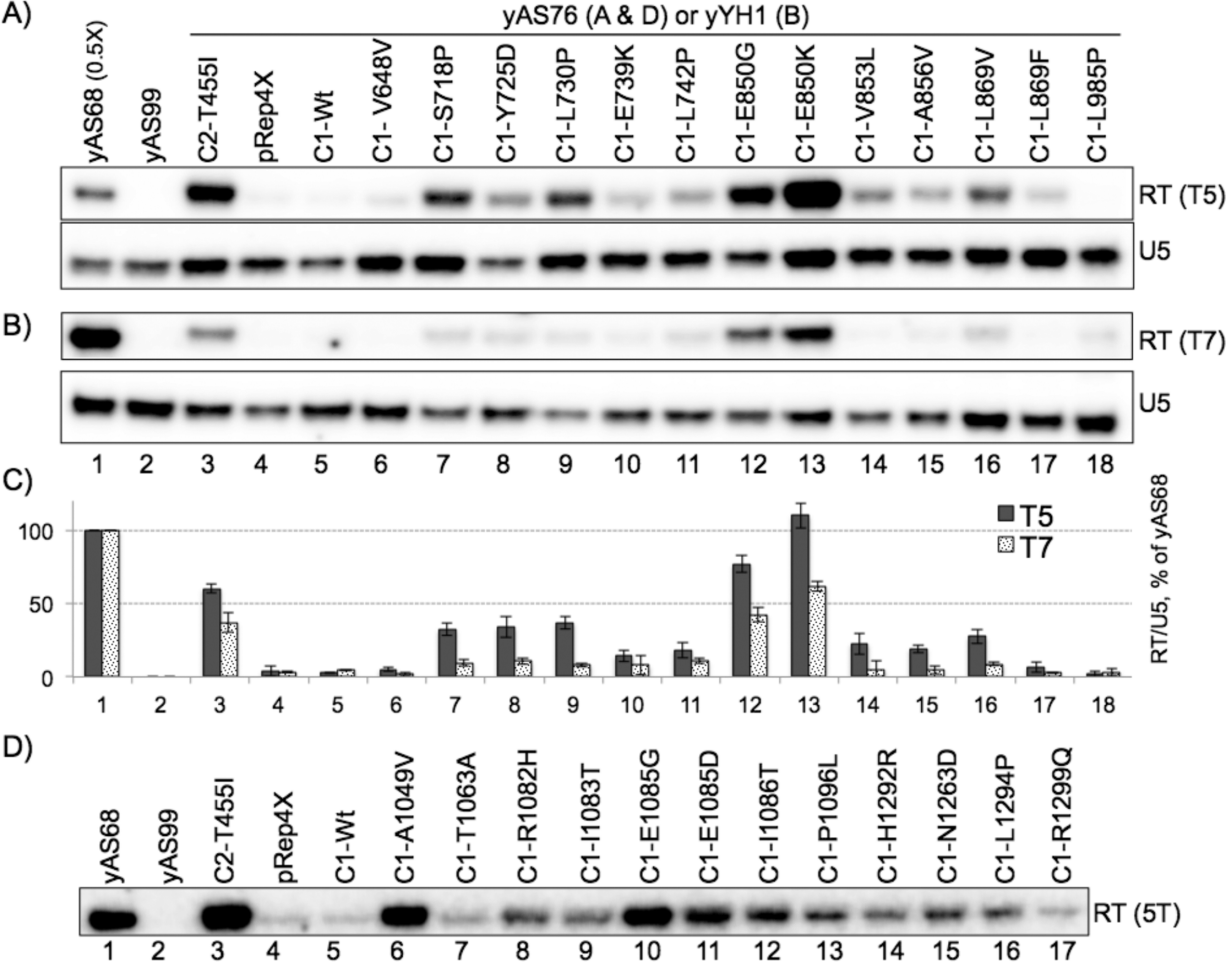
Validation of terminator readthrough *in vivo*. **A-D)** Northern blots of total RNA probed for the RNA species indicated to the right of the panels, produced by C1 mutant alleles in strain yAS76 (A & D) whose tRNA reporter gene has a 5T terminator (see Fig 5A) or in strain yYH1 (B) which has a 7T terminator; RT = readthrough transcript. Lanes 1 and 2 represent control strains yAS68 and yAS99 (see text). Error bars in (C) reflect RT/U5 from duplicate experiments plotted as % of yAS68 (right axis, see text).

Strain yAS76 carries a monomeric tRNA reporter gene with a 5T test terminator followed by a cRT sequence and a failsafe 8T terminator that the *in vitro* transcription data indicate is ~95% functional even in the strongest mutants (see Fig 5A; test terminator = 5T, and Fig 5B–5D). Thus, if RNAP III would read through the 5T test terminator it would synthesize a ~215 nt RT transcript. The cRT region is complementary to the upstream tRNA sequence and if transcribed will base pair with it to disrupt tRNA structure, stabilizing the ~215 nt RT transcript from processing [29, 35, 37] (Fig 7A upper; lower panel shows U5 RNA synthesized by RNAP II). The tRNA reporter gene in yAS68 (Fig 7A lane 1) is identical to yAS76 except with a 3T test terminator that produces ~100% RT transcript by terminating at the failsafe 8T terminator, with no mature tRNA produced (note that only 0.5X amount of yAS68 RNA was loaded, Fig 7A) [22, 29, 35]. The negative control strain, yAS99 (lane 2) lacks the tRNA reporter gene, while *rpc2*-mutant C2-T455I (lane 3) is a positive control for RT [29]. The yAS99, pRep4X vector, and Rpc1-Wt showed background levels of RT (Fig 7A, lanes 2, 4, 5).

In general agreement with their phenotypic strengths, the stronger mutants made more RT than the weaker (Fig 7A). This analysis for cellular RT transcripts was extended to yYH1 whose tRNA reporter allele has a 7T test terminator (Fig 7B). Only the two strongest, E850 mutants showed substantial RT (Fig 7B, lanes 12, 13).

We quantified the *in vivo* RT transcripts from the 5T and 7T terminators, calibrating the signal from yAS68 (3T test terminator) to 100% RT. C1-Wt and Rep4X showed very little RT as expected (Fig 7C, lanes 4–6) while the mutants produced more (lanes 7–18). Intriguingly, E850K reproducibly showed more RT than yAS68 (Fig 7C). As will be shown below, a substantial amount of E850K terminates at the 5T terminator and RT is only a fraction of its output. The Fig 7C quantitative data therefore suggest, and data in a later section demonstrate, that E850K produces more overall transcript per tRNA reporter gene than does endogenous wild type RNAP III (below).

Selected LOF mutants from lib-C were also assayed. Of these, A1049V and E1085 produced the most RT, consistent with their suppression phenotypes (Fig 7D).

### LOF mutants recognize and read through normal endogenous tRNA terminators

**Fig 8:**
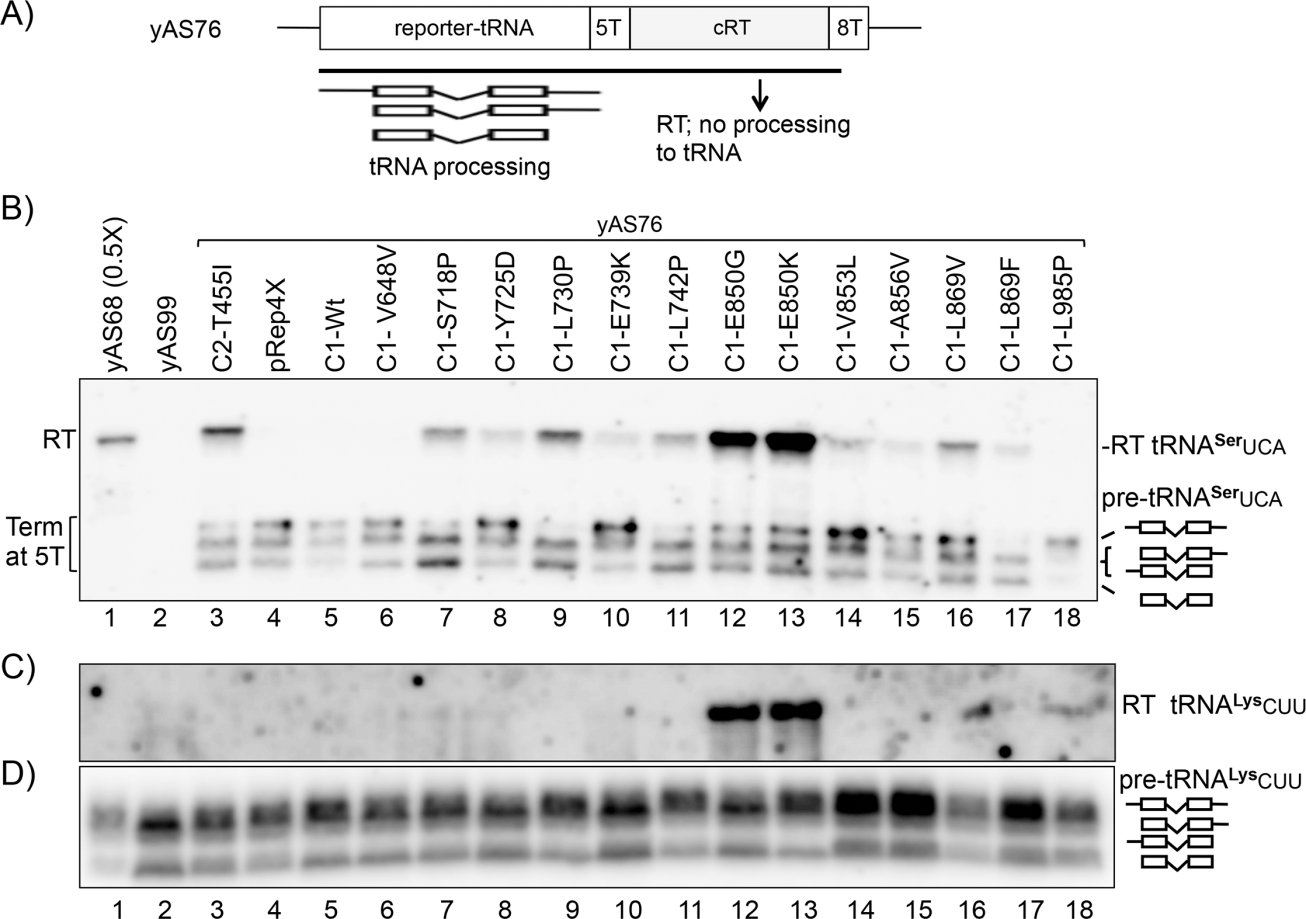
RNAP III C1 mutants recognize and read through normal terminators. **A)** Schematic of the tRNA reporter allele used for RT detection in strain yAS76, illustrating the transcripts produced from the 5T terminator and the 8T failsafe terminator. **B-D)** Northern blots. **B)** sup-tRNA intron-specific probe ("tRNA-Mser-int" see DNA oligos, Table 4); the RT transcript product resulting from termination at the failsafe 8T terminator, and pre-tRNA products resulting from termination at the 5T terminator, are indicated to the left. The intron-containing pre-tRNA processing species are indicated to the right. **C)** A readthrough-specific probe (equimolar mixture of three tRNA-LysCUU-RT oligo-DNAs, Methods) complementary to the region downstream of the natural terminator of tRNA-LysCUU gene, detects readthrough termination of endogenous tRNA gene (see text). **D)** A tRNALysCUU intron-specific probe (tRNA-LysCUU-int, Methods) of the same blot as in B (see text).

A probe complementary to the intron of a pre-tRNA can simultaneously detect transcripts released from the natural terminator that undergo rapid posttranscriptional processing to mature tRNA as well as 3'-extended transcripts that result from readthrough beyond the normal terminator (Fig 8A). The pre-tRNA species indicated to the right of Fig 8B reflect short-lived processing intermediates produced from the 5T terminator of the sup-tRNASerUCA gene that have been previously characterized [46, 47]. The yAS68 sup-tRNASerUCA allele with a 3T tract in place of the normal terminator produces no functional termination at this site as expected [22, 35] (Fig 8B, lane 1). As will be shown in a later section, the nascent pre-tRNA produced from the normal 5T terminator and the RT transcript from the failsafe 8T terminator exhibit different turnover rates (pre-tRNAs are processed more rapidly). Therefore, their relative amounts in Fig 8B do not simply reflect their relative termination efficiencies. In any case, the data show that C1-E850 (Fig 8B, lane 12 & 13) and other mutants accumulate variable amounts, in some cases as much or more of the pre-tRNA intermediates as the controls in lanes 4–5. In addition, we note that differences in the distribution of the three pre-tRNA bands among the lanes may reflect processing differences related to 3' oligo(U) length that can occur with termination mutants [29, 36, 37] and/or other effects associated with increased synthesis [48, 49]. The data indicate that termination at a natural 5T terminator is functional and substantial in the mutants, that some C1-mutants produce more pre-tRNAs than C1-Wt, and that the mutants also make significant RT, albeit to varying degrees.

Readthrough of some endogenous tRNA gene terminators may produce 3' extended RNAs that succumb to decay by nuclear surveillance [50]. We previously reported a RT transcript detectable by a probe complementary to the sequence downstream of the T5-C-T5 terminator of a tRNALysCUU gene on chromosome 2 [29]. The E850G and K mutants readily showed this RT transcript (Fig 8C). A probe to the intron in the nascent transcript from this gene and other copies of the pre-tRNALysCUU gene was used on the same blot (Fig 8D).

### C1 RT mutants produce more RNA transcript per tRNA reporter gene

**Fig 9:**
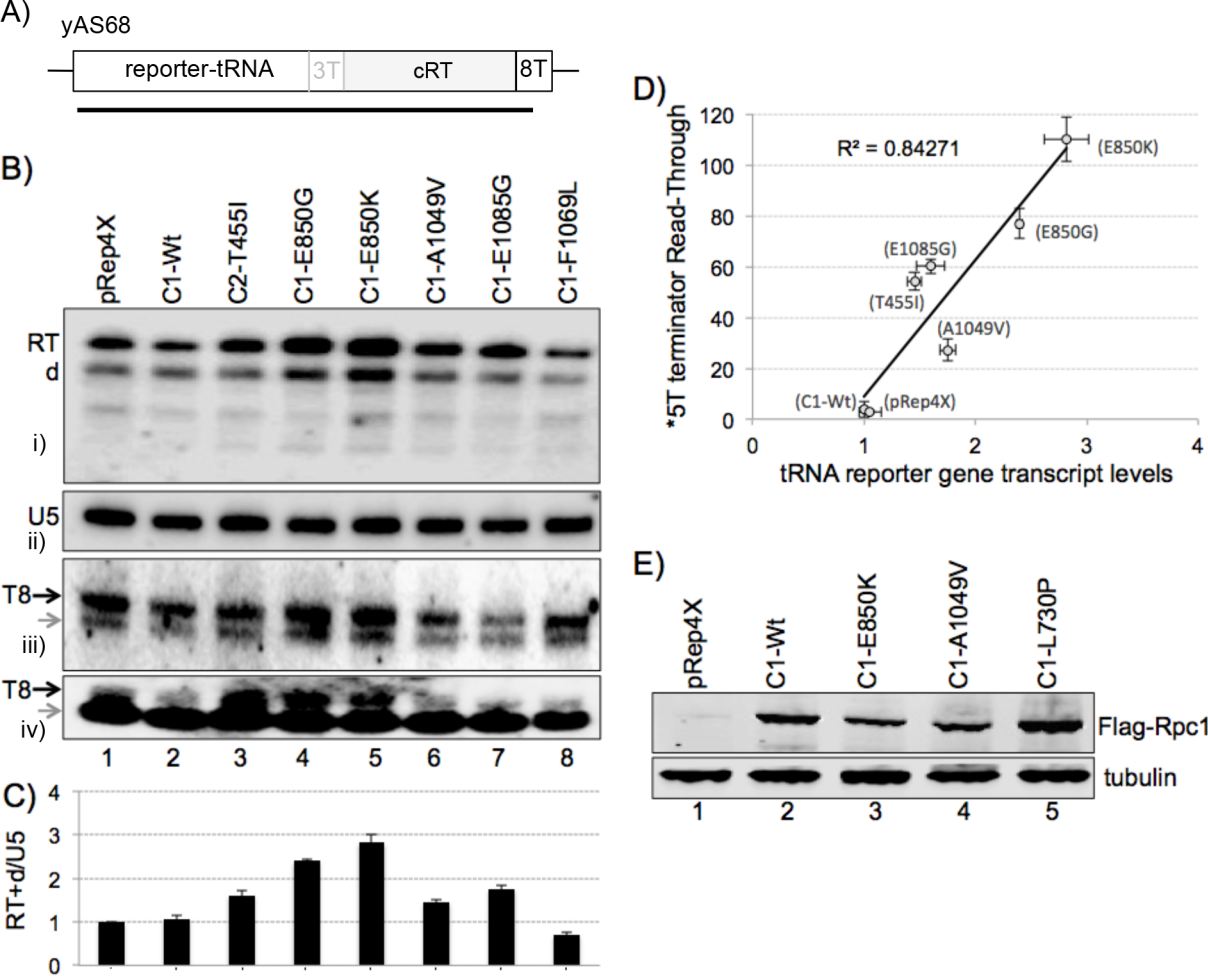
RNAP III C1 mutants increase RNA production from a tRNA gene. **A)** Schematic of the tRNA reporter gene in the strain yAS68 and the RT transcript produced from the 8T terminator. **B)** tRNA reporter gene readthrough (RT) transcript-specific probing of RNA from C1-mutants and controls as indicated above the lanes. Transcription of this tRNA reporter gene makes only RT transcripts and not the normal pre-tRNAs (upper panel, i, see text). The second panel (ii) shows probing of the same blot as in upper panel, for U5 snRNA. The next lower panel (iii) shows probing for the 3'-trailer of a nascent pre-tRNASerGCU whose endogenous single copy gene bears an 8T terminator (black arrow); the small grey arrow points to an intron-containing, 3'-trailer-containing intermediate. The lowest panel (iv) shows probing for the unique 3' trailer of a single copy nascent pre-tRNAValUAC whose gene does not contain an intron and ends with a T8 terminator (Black arrow); the small grey arrow points to the more abundant mature tRNAValUAC product of two genes that overlap with this probe. **C)** Quantification of data as in (A) from duplicate experiments calibrated by U5 RNA on the same blots; note that C1-F1069L, lane 8, is from a GOF mutant. **D)** RT transcript (Y-axis) from 5T terminator RT data plotted against amount of tRNA reporter gene output (X-axis) as in panel A; each represented by duplicate data sets reflected by error bars. The Y-axis represents a proxy for relative elongation rate (see text). **E)** Cells transformed with the pRep4X vector, C1-mutant or C1-Wt alleles carrying a Flag tag were subjected to immunoblotting for their Rpc1 protein.

As alluded to above, assessing RNA output by termination mutants was confounded by different termination efficiencies at T5 as well as potential differences in turnover of the RT transcript vs. the pre-tRNA transcripts. It is also known that in some conditions of elevated RNAP III activity, increased synthesis of pre-tRNA does not uniformly lead to increased levels of mature tRNAs [48, 49, 51]. To make comparisons more direct we used strain yAS68 whose tRNA reporter gene produces no nascent pre-tRNA but instead only the RT transcript of ~215 nt that does not undergo pre-tRNA processing [35] (lane 1 Fig 8B, Fig 9A). Thus, this reporter has two relevant characteristics, an 8T failsafe terminator that is functional in the mutants, and a primary transcript, RT that is not subjected to rapid tRNA processing. This reporter variably yields a degradation product, designated 'd' in the upper panel of Fig 9B, whose amount generally reflects the main band. The top panel of Fig 9B shows that the strongest mutants, C1-E850K and -E850G, produced more RT transcript than the other C1-mutants, C1-Wt or the pRep4X control. Quantification of data from replicate samples relative to U5 is shown in Fig 9C. C1-E850G and -E850K, produced 2-to-3 fold more RT RNA than pRep4X or C1-Wt, while C1-A1049V and -E1085G produced 1.5-to-1.8 fold more (Fig 9C). Importantly, the amount of RT RNA from RNAP III C1-Wt was comparable to the empty plasmid, pRep4X, providing evidence that ectopic expression of C1-Wt itself does not increase tRNA gene transcription output. This confirmed that transcript output per tRNA reporter gene is higher in the C1 LOF mutants relative to C1-Wt. Notably, we included one of the GOF mutants, F1069L, which in contrast to the LOF mutants showed decreased RNA production, at ~0.75 (Fig 9B lane 8, & Fig 9C).

As alluded to above, assessment of endogenous tRNA gene output in termination mutants is confounded by the fact that the amount of nascent pre-tRNA from the normal terminator may be diminished by read through. Also, as alluded to in the Introduction, there is reason to suspect that termination from long and short length oligo(T) terminators might plausibly differ in other ways. In *S*. *pombe* there are more tRNA genes terminated by oligo(T) tracts of 5 Ts than 6 or 7 Ts; only eight tRNA genes contain terminators of ≥8 Ts [52]. We wanted to see if such genes might show evidence of increased expression in the C1 LOF mutants. We examined the blot in Fig 9B with a probe containing sequence complementarity to the 3' trailer of a nascent pre-tRNASerGCU which has an intron and an 8T terminator (Fig 9B, panel iii). This probe detects the nascent transcript as indicated by the black arrow adjacent to panel iii of Fig 9B as well as an intron-containing, 3'-trailer-containing intermediate that lacks the 5' leader as indicated by the small grey arrow. Relative to the U5 loading control, the C1-E850 mutants showed more of the two trailer-containing, 8T-terminated transcripts than the C1-Wt. We next probed for the 3' trailer of a nascent pre-tRNAValUAC from a single copy gene with an 8T terminator, which does not have an intron (Fig 9B, panel iv, note that this probe overlaps with the mature tRNAValUAC produced from this and another gene, see figure legend). Again, the C1-E850 mutants showed more of the nascent 8T-terminated transcript than the C1-Wt. These data provide evidence to suggest that tRNA genes with long (8T) terminators reveal increased transcription in the C1 LOF mutants.

The cumulative Northern blot data provided substantial evidence to suggest that some C1 mutants produce more RNA than C1-Wt and this may be detected from some tRNA genes. Because the amount of readthrough of a 5T terminator likely reflects increased elongation rate as per kinetic coupling, the collective data further suggested that the LOF mutants produce more RNA because they are faster at elongation and/or other transitions in the transcription cycle. If kinetic coupling accounts for terminator RT, i.e., due to elongation, we wanted to ask if we might observe a correlation with transcript production by using the relative amount of readthrough by the mutants as a proxy for their relative elongation rates. We examined this by plotting tRNA reporter RT output (X-axis) against the previously determined amount of 5T terminator RT transcript (Y-axis), with duplicate data sets for both (Fig 9D). Although this approximation has limitations, a *R*^***2***^ coefficient of determination value of 0.84 indicates a robust correlation of terminator readthrough, serving as proxy for elongation rate, and tRNA reporter gene output.

We examined the levels of four FLAG-tagged C1 mutants by immunoblotting with anti-Flag antibody, using anti-tubulin for calibration (Fig 9E). The strongest phenotype mutants did not produce more C1 polypeptide than the C1-Wt (Fig 9E, compare lanes 3 & 4 with lane 2). These results indicated that higher tRNA reporter RT levels in C1-E850K and the other mutants examined is not due to higher levels of the C1-mutated proteins relative to C1-Wt. This confirmed previous functional data comparing C1-mutants and C1-Wt. Indeed, no correlation of transcription output with C1 expression levels is apparent, but appears instead to reflect their altered enzymatic properties.

### The RNAP III C1 mutant synthesizes more tRNA gene transcript *in vivo*

**Fig 10:**
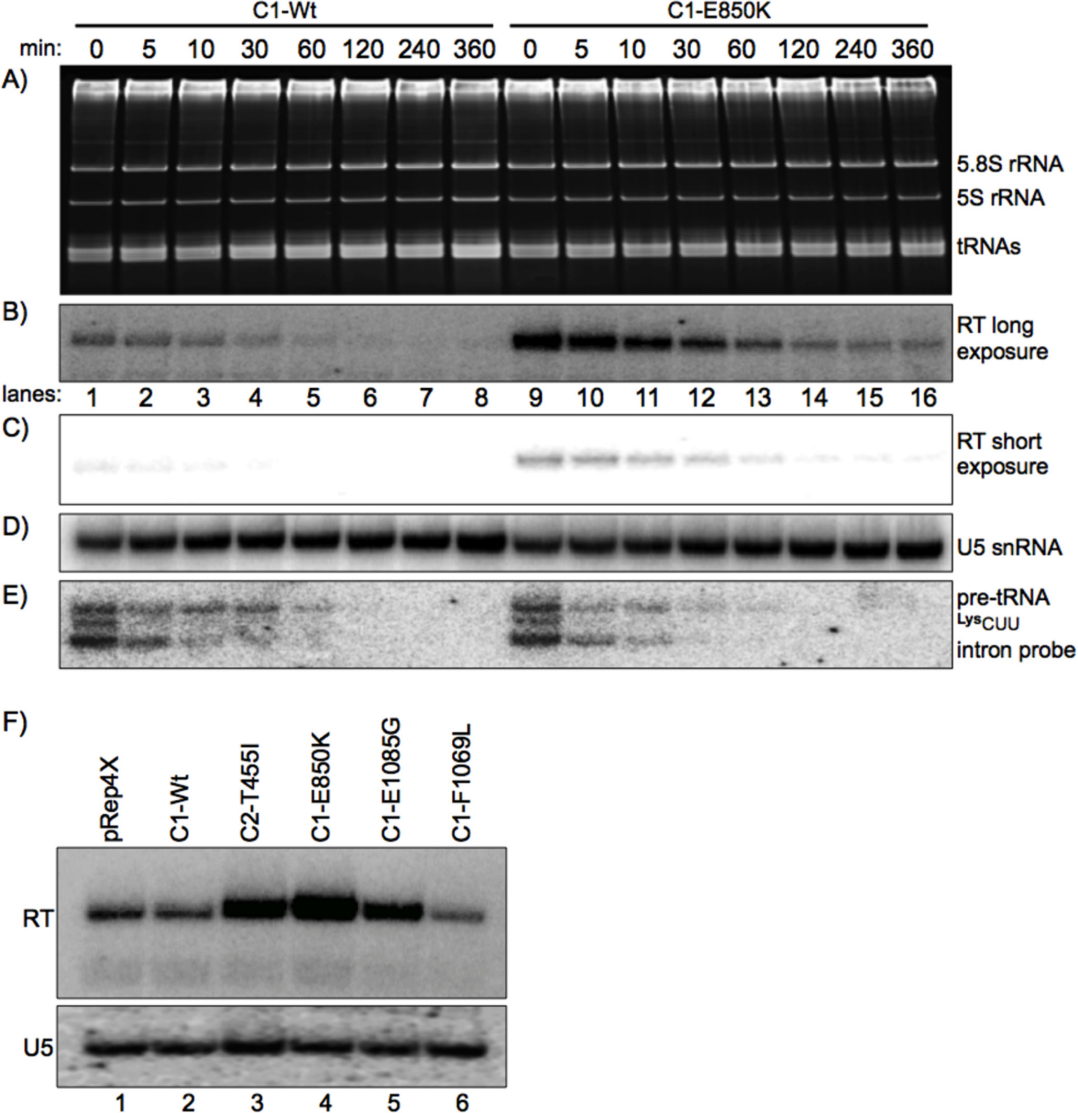
Synthesis rather than posttranscriptional RNA stability is increased by C1-mutants and unlinked to Maf1. C1-Wt and the multiple isolate, C1-E850K cells whose tRNA reporter gene (in yAS68 as in Fig 9B) makes only readthrough transcripts were treated with the transcriptional inhibitor, 1,10-phenanthroline (110-P), and RNA was purified from the cultures at the times indicated above the lanes. **A)** Ethidium bromide-stained gel that was blotted for sequential probing below as described in the text. **B & C)** Readthrough (cRT)-specific probe (tRNA-Mser-cRT; mix, Methods), long and short exposures; reveals nearly identical half-life of RT RNA in C1-Wt and C1-E850K, of about 20 minutes. **D)** Mature U5 snRNA probe. **E)** tRNALysCUU intron-specific probe reveals half-life of nascent pre-tRNALysCUU of about 5 minutes in C1-Wt and C1-E850K; note that C1-Wt and C1-E850K produce nearly equal amounts of this pre-tRNALysCUU (see also lanes 5 and 13 of Fig 7D, see text). **F)** A *maf1*-deletion strain, yKR101 carrying the same reporter tRNA gene as in Fig 9A and the C1-mutant or -Wt alleles indicated above the lanes were examined for the RT transcript (upper panel) and the U5 snRNA (lower).

The above data show that the mutants accumulate more RNA from the tRNA gene reporter than do the C1-Wt and other control. As noted above, some RNAP III termination mutants lead to alterations in the number of 3' terminal U residues on the nascent transcripts produced [29, 36, 37] [also see Figure 7 in 34]. This can be relevant because the La protein binds nascent RNAP III transcripts in a 3' oligo(U) length-dependent manner to stabilize some transcripts from 3' exonucleolytic decay [36]. In addition, increases in transcription by RNAP III can saturate some processing activities and effects on 3' processing can be conditional [48, 49]. Therefore, it was formally possible that the increase in RNA accumulation in the mutants was due to increased RNA stability rather than synthesis. The half-lives of nascent pre-tRNAs or the RT transcript from the tRNA reporter in *S*. *pombe* cells had not been previously determined. We examined this using 1,10-phenanthroline (110-P) which inhibits transcription in *S*. *pombe* [53, 54]. We compared the strongest, most frequently isolated mutant, C1-E850K, with C1-Wt in yAS68 by adding 110-P and isolating RNA at various times thereafter. The ethidium stained gel (Fig 10A) shows the high quality integrity of small stable RNAs over the course of the experiment. Fig 10B and 10C show long and short exposures of the blot made from this gel after probing for the RT transcript. Quantification of lanes 1 and 9 indicated ~4 fold more RT transcript in C1-E850K relative to C1-Wt at time zero relative to addition of 110-P, as compared to the stable RNAP II transcript, U5 RNA (Fig 10B and 10D). Comparison of lanes 1–8 and 9–16 of the RT RNA in Fig 10B & 10C revealed that it decayed at a similar rate in C1-Wt and C1-E850K mutant, with a half-life (50% remaining) of ~20 minutes (the constant low level observed at 120, 240 and 360 minutes in lanes 14–16 likely reflects leaky transcription). Thus, RT transcript decay is quite similar in C1-Wt and C1-E850K while abundance differs by ~4 fold. This provides strong evidence that the difference in RT levels is not due to its differential stability in the mutants, but it is instead because C1-E850K synthesizes more of the RNA than C1-Wt. An intron probe revealed that pre-tRNALysCUU intermediates have a shorter half-life than RT as expected, with the steepest decay occurring within five minutes (Fig 10E). We note that C1-Wt and C1-E850K produce near equal amounts of nascent pre-tRNALysCUU as also observed in lanes 5 and 13 of Fig 8D, whereas other C1 mutants vary in pre-tRNALysCUU production (e.g., compare lanes 13–16 of Fig 8B and8D).

### High output by the C1-mutants appears unrelated to the RNAP III repressor, Maf1

We reproducibly observed three to four fold more RNA production by the C1-E850K mutant than the C1-Wt (Figs 9A, 9B and 10B). This is a larger increase than expected based on their relative elongation rates observed *in vitro* (Fig 6) suggesting that the mutants RNAP III not only elongate faster than Wt, but may also initiate or reinitiate faster (Discussion). This suggested the possibility that the C1 mutations may have rendered these mutants refractory to the repressive effects of Maf1, the latter of which does not inhibit RNAP III elongation but inhibits its initiation [55–58]. If this was the case, we would expect that C1-Wt would be more activated by the absence of Maf1 than would the C1 mutants, and the differences in RT levels observed between C1-mutants and C1-Wt in *maf1*^***+***^ would be lessened by *maf1*-deletion. We examined RNA from the tRNA gene reporter that produces only the RT transcript in a *maf1*-deletion strain, yKR101 (Fig 10F). The pattern of relative amounts of RT transcript accumulation by C1 mutants, C1-Wt and pRep4X in the *maf1*-deletion strain (Fig 10F, upper panel) was generally similar to the *maf1*^***+***^ strain (Fig 9B). Quantification of the RT/U5 in Fig 10F showed that E850K produced about 4-fold more than C1-Wt, similar to the difference in *maf1*^***+***^ strains (Fig 9B and 9C). Thus, the relative amounts of RT produced by the LOF mutants examined and C1-Wt was not decreased by deleting *maf1*^***+***^. Reproducibly, the GOF mutant, C1-F1069L made less RT RNA than all of the others including C1-Wt (Fig 10F, compare lane 6 with lanes 1–5). These data suggest that the major positive effect of the C1-mutants on RT transcription output is not due to their ability to escape the repressive effects of Maf1.

## Discussion

Two genetic screens employing functional sup-tRNAs were used to identify residues in *S*. *pombe* Rpc1 that are important for termination by RNAP III. Mutagenesis was applied to full-length C1 to objectively identify the regions involved and the relative frequencies. Although this method can substitute all nucleotide bases to all others and produce a wide range of mutations [29, 59], it has limitations. Nonetheless, some positions were isolated numerous times and with multiple amino acids indicating a fairly thorough screen. It uncovered a wealth of mutants in the BH and TL, critical components of the elongation mechanism of all multisubunit RNAPs, as well as at the funnel and pore entrance, the latter likely outlining a path to the active center for the RNA 3' cleavage domain of C11 (below). Others have used mutagenesis to examine activities and functions of the BH and TL in RNAPs although those were targeted to specific positions rather than random throughout the full length largest subunit as was the approach here [42, 60–65]. Several of the mutants obtained in Rpc1 were at positions invariant or very highly conserved in other RNAPs that had been shown previously to either increase or decrease elongation rate, consistent with and supporting the kinetic coupling model of termination for RNAP III (below), and providing additional evidence that our screen was thorough.

### Elongation dynamics is a major governing determinant of RNAP III termination

Many mutants were obtained from the LOF screen which according to a kinetic coupling model would decrease pausing by increasing elongation rate. Experiments with purified enzymes using different templates and synchronized initiation confirmed that C1-E850K RNAP III, the most frequently isolated and strongest mutant, exhibited increased elongation rate relative to WT.

Twenty-five GOF mutants came from a screen for increased termination at 4T [35]. N491D and F494L are in the YNADFDGD aspartate triad motif found in all multisubunit RNAPs required for phosphodiester bond catalysis during elongation [41]. The homologous N in RNAP II contacts the 3'-OH of the incoming NTP [8]. Fifteen GOF mutations clustered in the TL tip with ten at 1067–1069; two of which were H1068Q. In RNAP II, the homologous His contacts the β-phosphate of the incoming NTP and is critical for catalysis [8]. These mutations are known to impair catalysis in other RNAPs and likely slow elongation by RNAP III. According to kinetic coupling this would increase pausing in the T-tract and enhance termination.

In summary, the data that emerges that strongly support kinetic coupling as a principal mechanism of termination control for RNAP III are several-fold: 1) GOF mutation of N in the invariant aspartate triad motif whose known function is in substrate binding and prior demonstrated mutation was shown to slow catalysis [41], 2) similarly, the GOF mutation H1068Q whose known function is in substrate binding and prior demonstrated mutation was shown to slow catalysis [8], 3) a high degree of concordance with prior data on multiple RNAP II TL mutation mutants that either increase or decrease elongation rate [42], and which was extended to the TL of RNAP I substituted with the RNAP III residue (Pol II: rpb1- E1103G; Pol I: rpa190-E1224G; Pol III: rpc1-E1085G) [62] and 4) *in vitro* transcription showing that purified RNAP III-E850K exhibited increased elongation rate relative to WT.

Although pausing is a prerequisite for termination, the degree to which elongation rate *per se* is a major governing determinant of termination appears to vary among RNAPs. For *E*. *coli* RNAP, slowing or stalling in a T-rich tract is not sufficient, as a RNA hairpin or Rho helicase is also required [66]. Slowing or stalling in oligo(T) is also insufficient for RNAP II as termination requires additional elements and activities [67]. Indeed, pausing without termination is a major point of regulation for RNAP II [68]. By contrast, sufficient pausing in oligo(T) leads to spontaneous termination by RNAP III [16, 22]. The only conditions we know that decrease the propensity of RNAP III for termination in oligo(T) is increased elongation rate, including by artificial removal of C53/37/11 [16, 28, 30]. The data here support the idea that RNAP III is unique in that elongation rate within oligo(T) is the major governing principal determinant of termination, with no known factors that offset this as part of a natural process or regulation.

### GOF and LOF mutants support a role for the RNA cleavage domain of C11 in termination

The TFIIS CTD and the C11 CTD are highly homologous and share most sequence identity in their hairpins [31] (Fig 3D). Examination of the TFIIS CTD structure placed into the RNAP III EC structure provided significant additional evidence of involvement of the RNA cleavage, acidic hairpin domain of C11 in termination. As described in the Results section, several C1 LOF mutations support involvement of the acidic hairpin CTD of C11 as a positive determinant of termination. The near identical sequences of the TFIIS and C11 acidic hairpins and observed close distances of known conserved interactions (Fig 3D), provide evidence that the TFIIS-CTD hairpin placement model is appropriate in this context and further support involvement of the C11-CTD and its RNA cleavage activity during termination [36], the requirement for a cleavage-active form of C11 for proper termination [16, 30] and for facilitated reinitiation [28].

In addition to support from the LOF mutations, we suspect that at least one of the GOF mutations, K780R, may work through the C11 acidic hairpin as well. The *S*. *cerevisiae* equivalent of K780 is K800 (Table 3). In the placement model, the basic side chain of K800 is positioned 5.7 Å from the acidic side chain of D at the DE tip of the hairpin. It is tempting to speculate that mutation of K with the longer side chain of R would place its basic moiety closer to and stabilizing the acidic tip, accounting for the GOF phenotype. The adjacent GOF mutation, S782P (Table 3, S802 in the RNAP III structure) may have similar effect by altering the backbone and allowing K780 closer access to the C11 acidic hairpin. The results suggest future experiments.

### An active center-driven model of RNAP III termination

The data suggest that the BH and TL LOF mutations promote faster catalysis and/or translocation, perhaps because the mutated motifs allow more dynamic movement. The data support an active center model of termination in which the oligo(rU:dA) hybrid and nontemplate Ts are critical. C53/37 slows elongation in oligo(T) [28, 69], possibly due in part to the ability of C37 to help recognize T3-T5 residues in the nontemplate DNA [16]. However, in the absence of C53/37, RNAP III-core terminates in the distal part of a 9T tract likely reflecting sensitivity of the active center to the inherent instability of a 9 bp oligo(rU:dA) hybrid [16, 30]. An appealing basis of this is that RNAP III exerts relatively loose grip on the hybrid relative to other RNAPs [17]. It was proposed that RNAP III can manage this because the relatively tight binding of downstream DNA by its cleft compensates during elongation [17]. Upon transcribing oligo(T), which is found only at the ends of class III genes, the exceptionally weak hybrid, oligo(rU:dA) [15] would decrease stability of the complex and together with nontemplate T effects on C37 [16], would cause termination.

In RNAP III, the clamp head of C1 comprises part of the downstream DNA binding cleft while the C1 jaw and C2 lobe comprise its other side [17]. C37 and C11 assemble on the peripheral side of the C2 lobe through multiple contacts [17]. Because the cleft forms a tight grip on downstream DNA [17], mutations in the C1 clamp head or jaw might have been expected from the termination screen. It is therefore notable that none of the 97 single mutations mutants mapped to these domains.

The CTD acidic hairpin of C11 is responsible for the RNA hydrolytic cleavage activity that is mediated by the RNAP III active center [31, 36, 70–72]. Analysis of the apoenzyme and EC structures of RNAP III indicate that while the NTD of C11 is fixed between C37, the lobe domain of C2 and the jaw of C1 at a similar position as the homologous NTDs of Rpb9 and A12.2 of RNAPs II and I respectively, the C11-CTD is flexible and temporally recruited to the catalytic center, homologous to TFIIS [17].

Mutations in the N- and C- terminal domains of C11 were independently isolated by two different screens for termination mutants [36, 37] one of which also identified a C-terminal 'hot-spot' region of C37 [29]. Crosslinking evidence indicate that parts of C37 extend to the active center [73, 74], including its hot-spot region which in the EC structure would appear to approach the single stranded nontemplate DNA in the active center, while another region connects to C11 more peripherally at the C2 lobe-C1 jaw region [17]. Examination of the EC structure also reveals that mutations in the C11-NTD mutants are close to C37 residues at the C1 jaw-C2 lobe, and the C37-R140 mutant mutation (*S*. *cerevisiae* C37 K185) lies close to C11 in the same area [17, 29, 37]. The collective data comprise good agreement of biochemical, genetic and physical evidence for functional connectivity of C37-C11-C53 from the active center to the jaw-lobe region of the cleft [17, 29, 31, 37, 44, 73, 74] that would probably be sensitive to movement of the CTD RNA cleavage domain of C11 [17]. Presumably, structural changes associated with termination-related activities in the active center may be transmitted to the cleft binding site this way. Potentially, transmission of allosteric information from the active center could contribute to termination by concerted action at the jaw side of the DNA binding cleft [6, 17].

### RNAP III active center mutations activate transcription of some tRNA genes

The relationship between transcription rate, termination and posttranscriptional processing is complex [48, 49, 51]. In some conditions of elevated RNAP III activity, increased synthesis of pre-tRNAs does not lead to much if any increase in mature tRNAs levels [48, 49, 51]. By monitoring transcripts produced *in vivo* from multiple of our tRNA reporter genes we noted a correlation of increased production with the degree of terminator readthrough among the mutants. The kinetic coupling model suggests that terminator readthrough reflects a correlation of elongation rate and transcript production. This further suggests that the mutants exhibit increased RNA synthesis because their BH and TL motifs are activated by mutations that promote faster translocation during elongation and perhaps other limiting steps during the transcription cycle.

The strongest C1-mutant exhibited 40% read through of a 5T terminator *in vitro* (Fig 5), and accumulated about 4-fold more reporter RNA *in vivo* than C1-Wt and the pRep4X control (Figs 9B, 9C, 10B and 10F) which can not be attributed to differences in RNA stability (Fig 10B). This increase relative to C1-Wt is higher than its nearly 2-fold increase in elongation rate relative to C1-Wt as approximated from analysis *in vitro* (Fig 6). It seems possible that the BH and TL mutations might allow the mutants to complete termination faster and/or to be reset for more efficient reinitiation, perhaps related to a faster rate of initial RNA synthesis upon reinitiation or more proficient promoter clearance. Consistent with this, TL mutations in Archaeal RNAP compromise initiation as reflected by deficiency in initial RNA synthesis [60], and RNAP II TL mutations may compromise or alter its responsiveness to TFIIB and TFIIF [61]. Indeed, yeast RNAP III is known to undergo repeated cycles of abortive initiation prior to promoter clearance that involves intrinsic RNA cleavage, and that it is rate limiting relative to transcription [75].

Genetic analysis of the C1 mutants was done in a Maf1-deleted strain. This indicated that as expected, their activating mutations do not render them refractory to the major inhibitory effects of the RNAP III general repressor, Maf1.

As alluded to above, the C1 BH and TL mutations may activate the transition between termination and reinitiation and/or promoter clearance. We note that termination in the T8 terminator of the reporter gene may contribute to this. As mentioned earlier, RNAP III has two potential modes, and this raises the possibility of differences in recycling potential after terminating in the proximal vs. distal parts of a T8 tract *in vivo*. For example, mutations that activate the mutants may do so better in long vs. short oligo(T) tracts. These matters raise suggestions for future investigations.

Terminators vary *in vivo*. Moreover, correlation of oligo(T) length and RNAP III occupancy [76] suggests that the terminator contributes to the stability of class III gene complexes [77, 78]. In this regard, we note that the strongest C1 mutants produced the most RT, bypassed the strongest terminator tested, 7T, longer than the average terminator in *S*. *pombe* [52, 78, 79], and were the most productive at tRNA reporter gene RT transcript output. These mutants produced the most RNA output when assayed using the tRNA reporter with the 8T terminator. They also showed more nascent pre-tRNA at the few endogenous tRNA genes that have an 8T terminator (Fig 9Biii, iv).

### Potential as a mechanism of RNAP III activation in cancer

A recent study reported three independent mutations in human RPC1 (POLR3A) associated with spontaneous cancers that were associated with autoantibodies to RNAP III [39]; The human *rpc1*-E1072Q occurred at the same invariant residue found in ten of our C1 mutants, E1085G/A/D. Of these, C1-E1085G was a strong phenotype and producer of substantial RT *in vivo* and transcript output. We also isolated ten mutants mutated at the next position, 1086 (Table 2). Given the association of activation of RNAP III and cancer [23, 24, 80], it should be suspected that the human *rpc1*-E1072Q mutation may have contributed to producing elevated tRNA and 5S rRNA in the cancer in which it occurred.

## Methods

### Mutagenesis

Deoxynucleotide analog-based mutagenic PCR was used to construct a C1 library as described [29], in three segments. For lib-A, primers C1-Xho-Fwd and C-NcoI-Rev, lib-B, C1-NcoI-Fwd and C1-BSTAP1-Rev, and lib-C, C1-BstAP1-Fwd and C1-Xma1-Rev were used (all DNA oligos used for this and other applications including northern blotting are listed in Table 4). The products were cloned into XhoI/XmaI of pRep4X and transformed into UltraMax DH5α-FT cells (30 plates). ~150,000 bacterial Transformants were scraped from the plates and plasmid DNA library prepared.

**Table 4:** DNA oligos.

C1-XhoI-Fwd	5’-TCAAGTTACTCGAGATGAAAGATCCTATTGATGATCAGG-3’
C1-NcoI-Rev	5’-ATTGTAGGGACCGCATACACATTCATTAAATCTTAAGGTACGCCATGGTC-3’
C1-NcoI-Fwd	5’ACTCCATAAGTTATCAATCATGGCTCACTTGGTAAAGGTACGACCATGGC-3’
C1-BstAp-Rev	5’TCAGTCACCCTGATAATGTTCTCAACAGATTTTCTTTTCGCAACGGGTGCA-3’
C1-BstAPI-Fwd	5’-ATGAATTTGCACCCGTTGCGAAAAGAAAATCT-GTTGAGAACA-3’
C1-XmaI-Rev	5’-TAGATCTACCCGGGTTAAGCCGTAATTTCCAATTGATG-3’
Rpc2_AscI_Fwd	5’- ATTAA GTAAGGCGCGCCATGGGGGTAAATACTGCC-3’
Rpc2_CHF_BglII-Rev	5’ATCGATAGATCTTCACTTATCGTCGTCATCCTTGTAATCGTGGTGATGGTGATGATGATACTTAAATTCGTCTTC -3’
C1-XhoI-Flag-Fwd	5’CAAGTCTCGAGATGGACTACAAAGACGATGACGACAAGAAAGATCCTATTGATGATCAGG-3’
TspR1 tailed template	5’ATCGCACTCACAGACCTGCAGTGAAGCGGACACAACCAGAGCAGGAAACTCCTAGCACAACTTTCATAGACAGCTCTGACTCTGGCTAGCTGACTTACACTTTACGATAGATGTACAGCTGCAGTCACTATGTCCGAGCTTTCAAGGAGTAGACTTCAGTCCTGCATGCTAGTCACCTTTCGGGCTCTGTCCGCGCAGGTTCAGATCCTGCTGGCGTTTGAAGATCTTCAGAGCTCGGTCAGAAAGGCCGGGCTCGGATTTGAGGTCTCCGTCACCAGCAGGATCCGAACCAATCAAGACTTTAATACAGGATTGAAGTCTAACTTTTTTTTTTTTGCCGCGAGTACCTGATCGCTCGATCCGTACGTACTAGGATCCTAGAGTCTTCCAGGAATCGGCATCCCGGGTACCATCG-3’
TspR1 tailed template (for walking)	5’ATCGCACTCACAGACCTGCAGTGAAGCGGACACAACCAGAGCAGGAATTTCCTAGCACAACTTTCATAGACAGCTCTGACTCTGGCTAGCTGACTTACACTTTACGATAGATGTACAGCTGCAGTCACTATGTCCGAGCTTTCAAGGAGTAGACTTCAGTCCTGCATGCTAGTCACCTTTCGGGCTCTGTCCGCGCAGGTTCAGATCCTGCTGGCGTTTGAAGATCTTCAGAGCTCGGTCAGAAAGGCCGGGCTCGGATTTGAGGTCTCCGTCACCAGCAGGATCCGAACCAATCAAGACTTTAATACAGGATTGAAGTCTAACTTTTTTTTTTTTGCCGCGAGTACCTGATCGCTCGATCCGTACGTACTAGGATCCTAGAGTCTTCCAGGAATCGGCATCCCGGGTACCATCG-3’
tRNA-LysCUU-RT	5’- TCTTCATACACTAGCATTTAATG-3’
	5’-CAGTATAAGGAAAAGTGGTGTAT-3’
	5’-ACATAGCAAGTGTGTTAACCATT-3’
tRNA-LysCUU-int	5’-CTTCTGATACCATTCGTAAGAGTC-3’
tRNA-Mser-cRT	5’-CCTGCTGGTGACGGAGACC-3’
	5’-TTCTAGTCTTGATTGGTTCA-3’
	5’-AGTTAGACTTCAATCCTG-3’
tRNA-Mser-int	5’-CATTAAATGACTAGAATACAGGATTGAAGTC-3’

### Strain construction

Suppressor-tRNA genes and *S*. *pombe* strains are listed in S1 Table. The Rpc2-C-His-FLAG tagged strain, yKR22 was constructed using pFA6a-kanMX6 vector. SpRpc2 was amplified with Rpc2-AscI-F and Rpc2-CHF-BglII-R (contains C-terminus His-FLAG sequence) primers and cloned into the pFA6a-kanMX6 in AscI and BglII site generating pFA6a-kanMX6-Rpc2. One kb sequences upstream and downstream of Rpc2 were also amplified from chromosomal DNA and cloned into pFA6a-KanMX6-Rpc2 in Xma1/AscI and PmeI/SacI sites respectively. For integration, the cloned fragment with kanamycin maker was amplified by PCR and integrated into yKR1 strain as described [81]. The *maf1-Δ* strain yKR101 was created as was the yNB1 *maf1-Δ* strain described previously [82] but carrying the tRNA reporter, pMSer-3T, with a 3T test terminator followed by cRT region and the 8T failsafe terminator: *h*− *leu1-32*::[*tRNAmSer3T-leu1+*] *ura4-D18 ade6-704 maf1Δ*::*KanMX6*.

### Plasmid construction

SpRpc1-Wt and selected mutants were cloned by PCR amplification from plasmids with forward primer (C1-XhoI-Flag-Fwd) and reverse primer (C1-XmaI–Rev). The forward primer contains 1X-Flag sequence at the N-terminus after the start codon. The PCR amplified fragments were gel purified, digested with XhoI and XmaI, and cloned into pRep4X at the corresponding sites.

### Isolation of C1 mutants

Libraries were transformed into *S*. *pombe* yKR1, plated on EMM lacking uracil with 10 mg/L adenine. Colonies showing suppression (white) were isolated, the plasmid was recovered and retransformed for conformation, and sequenced.

### Western blotting

For expression analysis, overnight cultures of Wt and mutants in EMM media lacking uracil were transferred to 50 ml and further grown to A600 of 0.8–1.0, washed with water, residual liquid aspirated and 200 μl of lysis buffer (50 mM HEPES (pH 7.3), 250 mM KCl, 5 mM EDTA, 2.5 mM DTT) was added. Glass beads (0.5 mm) were added and cells disrupted in mini-beadbeater-8 (Biospec) at 4 x 45 seconds keeping in ice for 1 min between intervals. Samples were then spun in tabletop centrifuge for 20 min at top speed, supernatant transferred to a new tube and protein concentration determined. 25 μg total protein was resolved in 4–12% Nu-PAGE gel (Life Technologies), transferred to nitrocellulose membrane. The blot was blocked for one hour with 5% non-fat milk in PBS. Monoclonal anti-flag (Sigma, #F1804) was used at 1:2000 in blocking buffer with 0.05% NP-40. Anti-Tubulin (Sigma, #T5168) was used at 1:5000. After one hour the blot was washed four times with PBS-NP-40. Appropriate secondary antibodies (LI-COR) of different fluorescence were used at 1:10,000 in 5% milk solution in PBS-NP-40 for one hour. The blot was washed with PBS-NP-40 four times and scanned using LI-COR Odyssey Clx system and the images processed with ImageStudioLite Software.

### Transcription extract

yKR1 transformed cells were grown in 2 liters of EMM lacking uracil until mid-log phase (OD600 = 2.0), harvested immediately, washed with ice-cold water, and as much liquid as possible aspirated leaving ~6 gm wet cell pellet which was frozen in dry ice. For S100 extract, the frozen cells were mixed with dry ice and shattered in a coffee grinder for 5 min, transferred to a beaker and 1 ml/gm cell pellet of extraction buffer (50 mM HEPES, 250 mM KCl, 5 mM EGTA, 1 mM EDTA) was added. The lysate was spun at 32K for 90 min. The supernatant, ~4 ml at 15–20 mg/ml was collected, aliquotted and immediately frozen on dry ice, leaving about 1/3 as the pellet.

### Promoter dependent transcription

Promoter dependent transcription was carried out by preincubating 3 μl of S100, 100 ng plasmid DNA containing Mser-tRNA genes with different test terminators (2T-8T) in transcription buffer (100 mM KCl, 20 mM HEPES pH 7.5, 7 mM MgCl_2_ and 3 mM DTT) at 25°C. After 30 min the NTPs were added at 0.5 mM ATP, CTP, UTP, and 25 μM GTP plus 5 μCi [α-32P]GTP (10 mCi/ml; 3000 Ci/mmol) in final volume of 25 μl. After another 30 min the reactions were quenched with 200 μl stop buffer (10 mM EDTA, 1% SDS, 25 μg/ml Proteinase K) and incubated for 10 min. Products were recovered by phenol-chloroform extraction, ethanol precipitation, resolved by 6% polyacrylamide-urea gel and visualized by a Typhoon 9400 phosphorImager (GE Healthcare). Image flies were analyzed using Multigauge Software (Fuji).

### Purification of epitope-tagged RNAP III for 3'-tailed template transcription

Briefly, 300 μl S100 extract (~6 mg protein) was mixed with 25 μl of EZview Red Anti-Flag-M2 Affinity Gel (Sigma, #F2426) and incubated at 4°C for 1 hr. The beads were spun, supernatant removed and beads washed 3X with 500 μl buffer (20 mM HEPES, 200 mM KCl, 1 mM EDTA). The beads with immobilized RNAP III was suspended in 100 μl transcription buffer. To this, tailed template and water was added, mixed by pipetting and distributed equally to multiple tubes for time course reactions, each carried out in 40 μl volume with 50 ng tailed template and other ingredients as indicated for promoter dependent transcription. The RNAP III stall-chase experiment was as above except using a tailed template lacking T in the first 22 nucleotides. The initial reactions were carried out in the absence of UTP for 2 min after which the reactions were made 50 μM UTP. Products were processed at the various time points thereafter as above.

For the excess template experiment, RNAP III was pull-down with EZview Red Anti-Flag-M2 Affinity Gel as above and incubated with excess of template (2 μg) in transcription buffer without NTPs for 10 min. After 10 min incubation at room temperature, the complex was washed with 500 μl of transcription buffer three times. After washing the complex was suspended into 300 μl transcription buffer and distributed into eight tubes for time course reaction. Time course was carried out with NTPs (50 μM for ATP, CTP, UTP and 25 μM GTP plus 5 μCi [α-^**32**^P]GTP and buffer as above.

### RNA stabilities were visualized

RNA stabilities were visualized by inhibiting cellular transcription in *S*. *pombe* with 1,10-Phenanthroline (Sigma Aldrich, Cat # 131377-5G) [53, 54]. Overnight cultures of C1-Wt and C1-E850K in yAS68 grown in EMM media lacking uracil were transferred to 500 ml fresh media and further grown to A600 of 0.5 OD. 1,10-Phenanthroline in DMSO was added at a final concentration of 350 μg/ml. At the times indicated thereafter 25 ml was withdrawn from the cultures, washed with water, spun down to aspirate residual water, and immediately frozen in dry ice. Total RNA was separated on 6% polyacrylamide-urea gel, and processed for northern blotting as described.

### Northern blotting

Total RNA was isolated using hot phenol. RNA from control strains and mutants were separated on 6% or 10% polyacrylamide-urea gel, and transformed to a nylon membrane using the IBlot apparatus (Invitrogen, Grand Island, NY). Membranes were UV cross linked, vacuum dried at 80°C and hybridized with RNA specific DNA oligos as indicated and listed in Table 4. After washing, blots were exposed to phosphorimager screen, scanned by a Typhoon 9400 phosphorImager and quantified using Multigauge software (Fuji).

## Supporting Information

S1 Fig Results of site directed mutagenesis of C1 E850. (PDF)

S2 Fig Establishment of factor-independent, 3'-tailed template, transcription elongation assay for purified RNAP III. **A)** Schematic of 3'-tailed template used for promoter-independent transcription by FLAG-purified RNAP III. **B)** Extract from yAS99 which has no tagged subunit served as a control (lanes 1–10). RNAP III purified from *S*. *cerevisiae* produced the FL band as expected (lanes 23–24, asterisk). Purified *E*. *coli* RNAP transcribed through the 12T tract to produce run-off (RO) as expected (lanes 27–28). *S*. *pombe* RNAP III produced FL RNA from yKR22 (lanes 19–20). Additional specificity features detailed above the lanes established a promoter-independent transcription termination assay.
(PDF)

S1 Table Strains used in this study. (PDF)
