# Supplementary material for: Active Center Control of Termination by RNA Polymerase III and tRNA Gene Transcription Levels In Vivo
Source: PLoS Genet. 2016 Aug 12;12(8):e1006253. doi: 10.1371/journal.pgen.1006253 (PMC4982682; doi:10.1371/journal.pgen.1006253)
Supplement: S1 Fig — (PDF) [file pgen.1006253.s001.pdf]

Supplemental Figure S1. Rijal and Maraia

Site directed mutation at E850 position of Rpc1

yKR1 (T5 terminator)

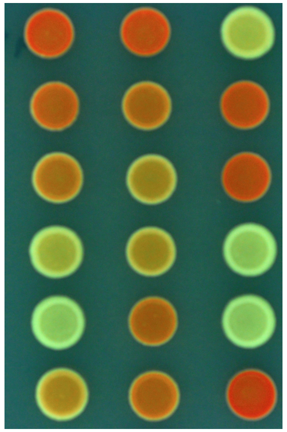

EMM Ura-, Ade10

Rep4X	Rpc1 wt	Rpc2-T455I
E850L	E850I	E850F
E850V	E850C	E850Y
E850A	E850T	E850G
E850S	E850D	E850K
E850N	E850H	E850P

yJI1 (T6 terminator)

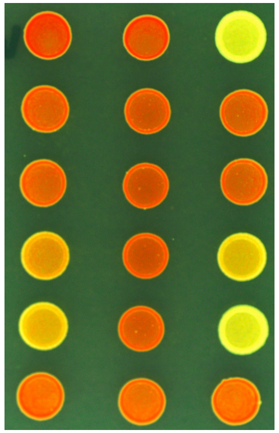

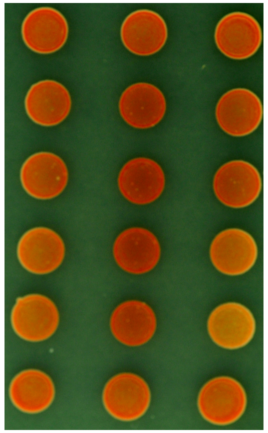

EMM Ura-, Ade10 + Thiamine

Mutation of E:
-G, S, and K has stronger suppression
-A, C,N and T has weak suppression
-Bulky group (F, Y) or Proline has no suppression

Mutation not made: R, W, M, Q
