# Supplementary figures and images for: Active Center Control of Termination by RNA Polymerase III and tRNA Gene Transcription Levels In Vivo
Source: PLoS Genet. 2016 Aug 12;12(8):e1006253. doi: 10.1371/journal.pgen.1006253 (PMC4982682; doi:10.1371/journal.pgen.1006253)

|    |    |    |    |    |                                       |    |    |    |    |                             |    |    |    |  |                     |
|----|----|----|----|----|---------------------------------------|----|----|----|----|-----------------------------|----|----|----|--|---------------------|
| +  |    |    |    |    | Purified<br>S. cerevisiae<br>RNAP III |    |    |    |    | Purified<br>E. Coli<br>RNAP |    |    |    |  | ssDNA mol wt marker |
| -  | -  | -  | -  | -  |                                       |    |    |    |    |                             |    |    |    |  |                     |
| -  |    | +  | +  | +  | -                                     | -  | +  | +  | -  | -                           | +  | +  |    |  |                     |
| -  | +  | -  | -  | -  | -                                     | +  | -  | -  | -  | +                           | -  | -  |    |  |                     |
| 60 | 60 | 0  | 30 | 60 | 60                                    | 60 | 60 | 30 | 60 | 60                          | 60 | 30 |    |  |                     |
| 16 | 17 | 18 | 19 | 20 | 21                                    | 22 | 23 | 24 | 25 | 26                          | 27 | 28 | mw |  |                     |

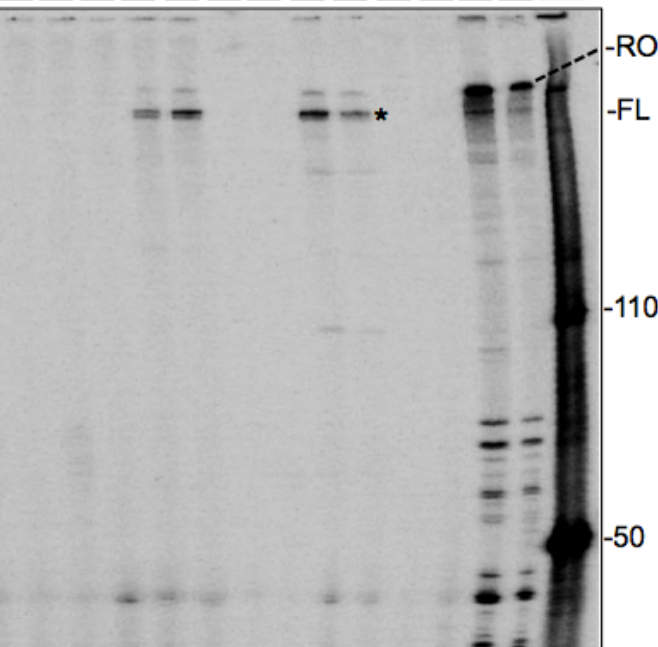

Supplement: S2 Fig — A) Schematic of 3'-tailed template used for promoter-independent transcription by FLAG-purified RNAP III. B) Extract from yAS99 which has no tagged subunit served as a control (lanes 1–10). RNAP III purified from S. cerevisiae produced the FL band as expected (lanes 23–24, asterisk). Purified E. coli RNAP transcribed through the 12T tract to produce run-off (RO) as expected (lanes 27–28). S. pombe RNAP III produced FL RNA from yKR22 (lanes 19–20). Additional specificity features detailed above the lanes established a promoter-independent transcription termination assay. (PDF) [file pgen.1006253.s002.pdf]
