# Supplementary material for: Active Center Control of Termination by RNA Polymerase III and tRNA Gene Transcription Levels In Vivo
Source: PLoS Genet. 2016 Aug 12;12(8):e1006253. doi: 10.1371/journal.pgen.1006253 (PMC4982682; doi:10.1371/journal.pgen.1006253)
Supplement: S1 Table — (PDF) [file pgen.1006253.s003.pdf]

<u>Table S1.</u> *Schizosaccharomyces pombe* strains used in this stud

Strain name	Genotype	Reference
yAS99	h- ade6-704 ura4-D18 leu1-32	[1-3]
yYH1	h- ade6-704 ura4-D18 leu1-32::[tRNAmSer7T-leu1$^+$]	[4]
yAS68	h- ade6-704 ura4-D18 leu1-32::[tRNAmSer3T-leu1$^+$]	[5]
yAS70	h- ade6-704 ura4-D18 leu1-32::[tRNAmSer4T-leu1$^+$]	
yAS76	h- ade6-704 ura4-D18 leu1-32::[tRNAmSer5T-leu1$^+$]	[6]
yKR1	h- ade6-704 ura4-D18 leu1-32::[DRT5T-leu1$^+$]	[6]
yJI1	h- ade6-704 ura4-D18 leu1-32::[DRT6T-leu1$^+$]	[5]
yKR22	h- ade6-704 ura4-D18 leu1-32::[DRT5T-leu1$^+$] rpc2::(rpc2-FH-KanMX6)	this study
yKR101	h– leu132::[tRNAmSer3T-leu1+] ura4-D18 ade6-704 maf1Δ::KanMX6. (made from yAS68)	this study

[1]Huang Y, Bayfield MA, Intine RV, Maraia RJ. Separate RNA-binding surfaces on the multifunctional La protein mediate distinguishable activities in tRNA maturation. Nat Struct Mol Biol. 2006 Jul;13(7):611-8.

[2]Iben JR, Epstein JA, Bayfield MA, Bruinsma MW, Hasson S, Bacikova D, et al. Comparative whole genome sequencing reveals phenotypic tRNA gene duplication in spontaneous Schizosaccharomyces pombe La mutants. Nucleic Acids Res. 2011 Feb 11;39:4728-42.

[3]Intine RVA, Sakulich AL, Koduru SB, Huang Y, Pierstorrf E, Goodier JL, et al. Control of transfer RNA maturation by phosphorylation of the human La antigen on serine 366. Mol Cell. 2000;6:339-48.

[4]Huang Y, Intine RV, Mozlin A, Hasson S, Maraia RJ. Mutations in the RNA Polymerase III Subunit Rpc11p That Decrease RNA 3' Cleavage Activity Increase 3'-Terminal Oligo(U) Length and La-Dependent tRNA Processing. Mol Cell Biol. 2005;25(2):621-36.

[5]Iben JR, Mazeika JK, Hasson S, Rijal K, Arimbasseri AG, Russo AN, et al. Point mutations in the Rpb9-homologous domain of Rpc11 that impair transcription termination by RNA polymerase III. Nucleic Acids Res. 2011;39:6100-13.

[6]Rijal K, Maraia RJ. RNA polymerase III mutants in TFIIFα-like C37 cause terminator readthrough with no decrease in transcription output. Nucleic Acids Research. 2013;41:139-55.
